# Molecular Mechanisms by Which a *Fucus vesiculosus* Extract Mediates Cell Cycle Inhibition and Cell Death in Pancreatic Cancer Cells

**DOI:** 10.3390/md13074470

**Published:** 2015-07-20

**Authors:** Ulf Geisen, Marion Zenthoefer, Matthias Peipp, Jannik Kerber, Johannes Plenge, Antonella Managò, Markus Fuhrmann, Roland Geyer, Steffen Hennig, Dieter Adam, Levent Piker, Gerald Rimbach, Holger Kalthoff

**Affiliations:** 1 Division of Molecular Oncology, Institute for Experimental Cancer Research, Medical Faculty, CAU, University Hospital Schleswig-Holstein, 24105 Kiel, Germany; E-Mails: ugeisen@email.uni-kiel.de (U.G.); jannik-kerber@t-online.de (J.K.); 2 CRM, Coastal Research & Management, 24159 Kiel, Germany; E-Mails: m.zenthoefer@web.de (M.Z.); steffen.hennig@crm-online.de (S.H.); lpiker@oceanbasis.de (L.P.); 3 Division of Stem Cell Transplantation and Immunotherapy, Department of Internal Medicine II, University Hospital Schleswig-Holstein, 24105 Kiel, Germany; E-Mail: m.peipp@med2.uni-kiel.de; 4 Institute of Immunology, University Hospital Schleswig-Holstein, 24105 Kiel, Germany; E-Mails: Johannes.plenge@gmx.de (J.P.); dadam@email.uni-kiel.de (D.A.); 5 Department of Biology, University of Padua, 35131 Padua, Italy; E-Mail: manago.antonella@gmail.com; 6 Numares AG, 93053 Regensburg, Germany; E-Mails: markus.fuhrmann@numares.com (M.F.); Roland.Geyer@numares.com (R.G.); 7 Institute of Human Nutrition and Food Science, Christian-Albrechts University of Kiel, 24118 Kiel, Germany; E-Mail: rimbach@foodsci.uni-kiel.de

**Keywords:** algae, *Fucus vesiculosus*, pancreatic, cancer, cell cycle inhibitors, autophagy, proliferation

## Abstract

Pancreatic cancer is one of the most aggressive cancer entities, with an extremely poor 5-year survival rate. Therefore, novel therapeutic agents with specific modes of action are urgently needed. Marine organisms represent a promising source to identify new pharmacologically active substances. Secondary metabolites derived from marine algae are of particular interest. The present work describes cellular and molecular mechanisms induced by an HPLC-fractionated, hydrophilic extract derived from the Baltic brown seaweed *Fucus vesiculosus* (Fv1). Treatment with Fv1 resulted in a strong inhibition of viability in various pancreatic cancer cell lines. This extract inhibited the cell cycle of proliferating cells due to the up-regulation of cell cycle inhibitors, shown on the mRNA (microarray data) and protein level. As a result, cells were dying in a caspase-independent manner. Experiments with non-dividing cells showed that proliferation is a prerequisite for the effectiveness of Fv1. Importantly, Fv1 showed low cytotoxic activity against non-malignant resting T cells and terminally differentiated cells like erythrocytes. Interestingly, accelerated killing effects were observed in combination with inhibitors of autophagy. Our *in vitro* data suggest that Fv1 may represent a promising new agent that deserves further development towards clinical application.

## 1. Introduction

Pancreatic cancer has an overall five-year survival rate of 7.2% (in 2010) [1] and the only curative treatment option—being limited to less than 20% of the patients—is resection of the tumor by surgery. However, also patients with resection are dying because of tumor recurrence or metastasis development [2]. Pancreatic cancer is one of the few tumor entities for which the incidence and mortality rate is even predicted to increase in the next years [1,3]. Chemotherapeutic treatment regimens for pancreatic cancer have improved within the last years, but prolonged lifetime is limited to only a few months [2]. New therapeutic options are urgently needed.

Brown algae might represent an invaluable source for identifying new therapy agents since they are rich in sulfated polysaccharides and secondary plant metabolites like fucoidans, fucoxanthin or phlorotannins [4,5,6]. Besides antioxidant activity and beneficial effects on cardiovascular disease, these substances were shown to exhibit anti-cancerous properties [4,5,7,8,9,10]. Fucoidans are known to inhibit proliferation in tumor cells [11,12]. For fucoxanthin and phlorotannins, anti-proliferative and apoptotic effects are known [13,14,15,16]. However, the particular mechanisms are not yet unraveled.

In the present work, we systematically investigated the effect of a purified acetonic extract of the Baltic brown seaweed *Fucus vesiculosus* (called Fv1) on human cancer and non-malignant cell lines. We studied its effects on the gene expression and protein level and our analyses suggest cell cycle control mechanisms as the major mode of action.

## 2. Results

### 2.1. Influence of Fv1 on Viability of Cancer Cells

**Figure 1:**
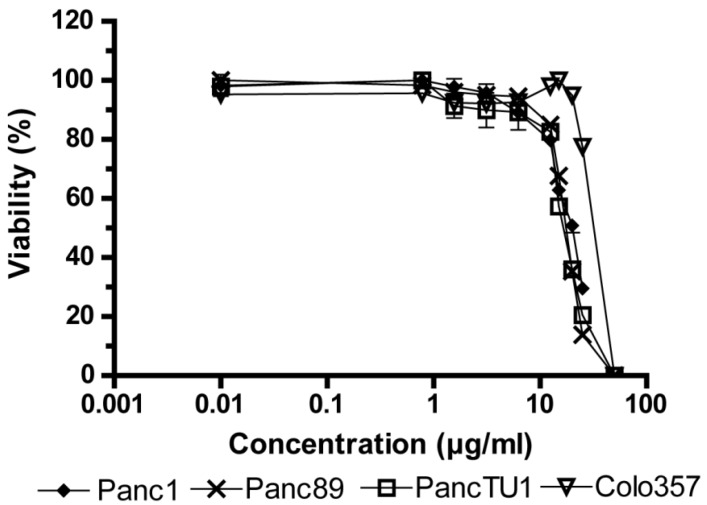
Inhibition of cell viability by *Fucus vesiculosus* (Fv1) in different cancer cell lines. 5 × 10^3^ cells were seeded in 96 well plates and treated with Fv1 or dimethyl sulfoxide (DMSO) as control (0.15%) after 24 h. After 72 h treatment, an AlamarBlue viability assay was performed. Values are presented as % of control; concentrations are shown using a logarithmic scale. Raw data are shown in Supplementary Table S1. *n* = 4.

**Figure 2:**
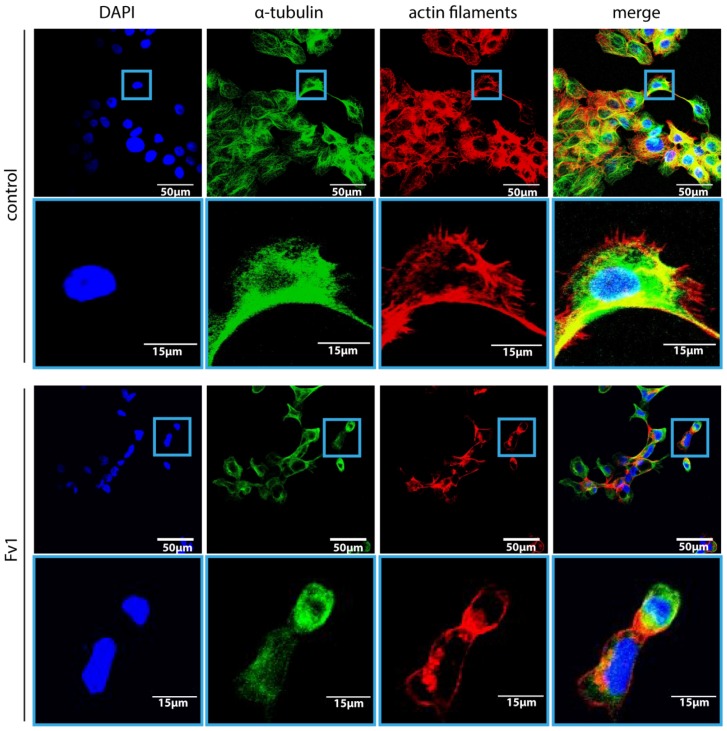
Fv1 leads to decreased cell numbers and to morphological alterations. Panc89 cells were seeded on coverslips and treated with Fv1 (10 µg/mL) or DMSO (0.125%)-containing cell culture medium. After 24 h, the cells were stained with an α-Tubulin antibody (2nd antibody: α-mouse, Alexa 488-coupled) and with phalloidin (Alexa 546-coupled) for actin cytoskeleton staining. The coverslips were mounted using a DAPI-containing mounting medium. Representative pictures were taken with a Zeiss CLSM. Two magnifications are shown.

First, we analyzed the effect of Fv1 on the viability of tumor cells. Fv1 inhibited the growth of different tumor cell lines significantly (Figure 1). The EC50 (effective half maximal concentration) values of Fv1 range between 17.35 µg/mL for PancTU1 (95% CI: 16.74–17.99), 17.5 µg/mL for Panc89 (95% CI: 17.24–17.77), 19.23 µg/mL for Panc1 (95% CI: 18.52–19.98) and 28.9 µg/mL for Colo357 (95% CI: 22.71–32.11). Morphologically, Fv1-treated cells exhibited more spindle-like cells, observed with staining of actin and tubulin (Figure 2). Treated cells changed their microfilamental structures. Moreover, they rather grew in a solitary way and did not form dense epithelial structures like untreated cells do. Figure 2 shows one representative experiment with Panc89 pancreatic ductal adenocarcinoma (PDAC) cells.

**Figure 3:**
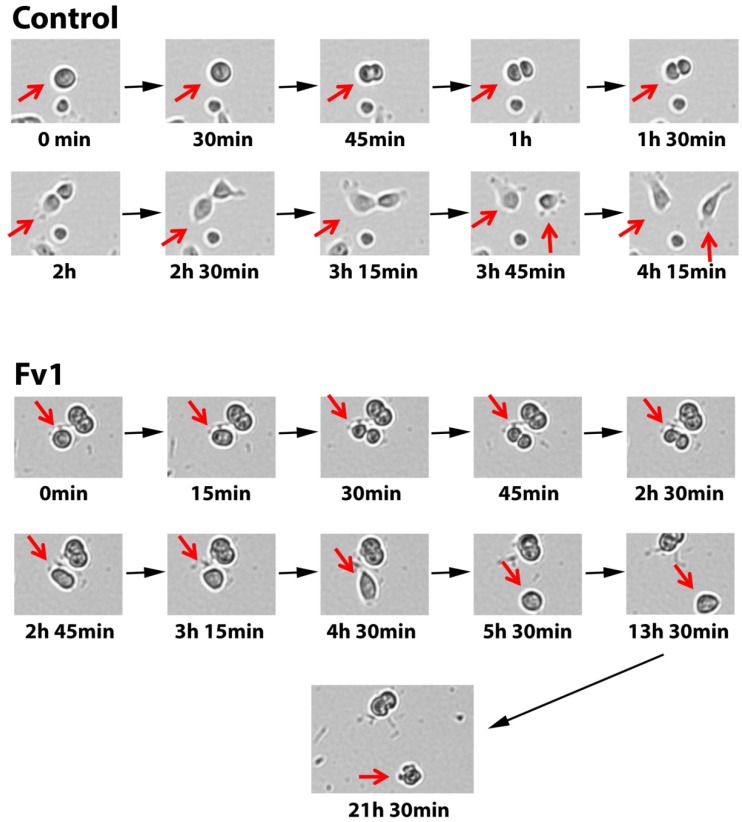
Fv1 inhibits mitosis. Human pancreatic ductal epithelial (HPDE) cells were treated with Fv1 in a lethal dose (50 µg/mL) and observed using the JuLI Br Live Cell Analyzer. Pictures were taken every 15 min automatically for 24 h. Representative pictures show one single cell undergoing mitosis.

To get more insight into the time-dependent morphological changes induced by Fv1, live cell imaging was performed by taking microscopic images every 15 min. While untreated cells divided normally, we observed many Fv1-treated cells entering mitosis, showing a cleaving furrow but then the cells rounded up and died. Often, cell fragmentation was observed several hours later. Representative images of this process are given in Figure 3. 

### 2.2. Effect of Fv1 on Cell Cycle and Cell Cycle Inhibitors

To elucidate the molecular mechanism mediated by Fv1 in more detail, we performed large scale gene expression profiling on over 40,000 transcripts using Agilent arrays, comparing Fv1-treated with untreated cells. The expression of many genes was significantly changed (Table 1A). Fv1 regulated about 10-fold less genes in Colo357 cells than in the cell lines Panc1, Panc89, PancTU1 and HPDE. 157 genes were found to be commonly deregulated in the treated cell lines Panc89, Panc1 and PancTU1. Many of these genes are involved in cell cycle control, DNA repair and also in inflammation and cancer (Table 1B). Because of these findings, we focused on cell cycle regulating pathways. Interestingly, the cell cycle inhibitor p57 was induced in three cancer cell lines (Panc1, Panc89, PancTU1). Accordingly, some downstream targets that are inhibited by p57 were suppressed (Cyclin E2, CDC45, CDC7, CDC25A, E2F1, PCNA, see Table 1C and Supplementary Figure S1 for the pathway graphic). Furthermore, the expression of the upstream regulator “tumor protein 53 inducible protein” TP53INP1 was increased. In addition, the expression of cell division cycle protein 20 (CDC20) which activates the anaphase promoting complex (APC) [17], was decreased in three cell lines (Panc1, PancTU1, Panc89, see Table 1C). This led us to the suggestion that Fv1 induces a cell cycle arrest.

**Table 1:** Fv1 regulates pathways involved in DNA-replication and cell cycle cells were cultured and incubated with Fv1 for 24 h. Whole cell lysates were produced by pooling attached cells and detached cells in the supernatant. RNA was isolated, reverse-transcribed and hybridized to Agilent 40K chips. After hybridization, data were normalized according to the analysis standards of SourceBioscience (Berlin). (**A**) Number of significantly regulated genes after Fv1 treatment. Criteria of significance were a *p*-value < 0.05 and a log fold change (lfc) >1 or <−1; (**B**) Collectively regulated genes of the cell lines Panc1, PancTU1 and Panc89 were analyzed using the DAVID (Database for Annotation, Visualization and Integrated Discovery) functional annotation clustering tool and KEGG (Kyoto Encyclopedia of Genes and Genomes) pathway. The most probable pathways, according to DAVID analysis, are shown. All experiments were performed in biological triplicates; (**C**) Gene expression data of single genes which are involved in the KEGG pathway of cell cycle regulation are shown with log fold changes (log 2) and bonferroni-corrected *p*-values.

**A**									
**Cell Line**				**Number of Regulated Genes**					
Panc1				3951					
Panc89				3909					
HPDE				2614					
Colo357				200					
PancTU1 6 h				340					
PancTU1 24 h				2930					
**B**									
**Term**			**Count**			**% of Pathway Genes**		***p*-Value (Benjamini-Hochberg-Corrected)**	
DNA replication			8			6.25		0.0000012	
Cell cycle			11			8.59		0.0000034	
Base excision repair			5			3.91		0.0045238	
Terpenoid backbone biosynthesis			4			3.13		0.0038877	
Oocyte meiosis			5			3.91		0.1670225	
Lysosome			5			3.91		0.1705743	
Pyrimidine metabolism			4			3.13		0.3412000	
Nucleotide excision repair			3			2.34		0.3292213	
**C**										
**Target ID**	**Panc1**		**PancTU1**			**Panc89**		**Gene Symbol**	**Gene Name**	
	**Lfc**	***p*-Value**	**Lfc**		***p*-Value**	**Lfc**	***p*-Value**			
NM_000076	2.0364	0.0326	1.0465		0.0261	2.7013	0.0347	CDKN1C	cyclin-dependent kinase inhibitor 1C (p57, Kip2)	
NM_057749	−1.9238	0.0470	−1.9484		0.0003	−4.3172	0.0050	CCNE2	cyclin E2	
NM_003504	−1.5647	0.0243	−1.3113		0.0054	−3.5616	0.0005	CDC45	cell division cycle 45 homolog (*S. cerevisiae*)	
NM_003503	−1.3615	0.0132	−1.5369		0.0012	−3.7463	0.0081	CDC7	cell division cycle 7 homolog (*S. cerevisiae*)	
NM_001789	−1.9897	0.0263	−1.8791		0.0001	−4.5741	0.0359	CDC25A	cell division cycle 25 homolog A (*S. pombe*)	
NM_005225	−1.3352	0.0197	−1.3330		0.0004	−3.2981	0.0415	E2F1	E2F transcription factor 1	
NM_002592	−1.3851	0.0047	−1.1083		0.0018	−2.9858	0.0056	PCNA	proliferating cell nuclear antigen	
NM_033285	2.2445	0.0023	3.9746		0.0052	5.6604	0.0427	TP53INP1	tumor protein p53 inducible nuclear protein 1	
NM_001255	−1.1762	0.0036	−1.0547		0.0160	−3.2856	0.0221	CDC20	cell division cycle 20 homolog (*S. cerevisiae*)	

**Figure 4:**
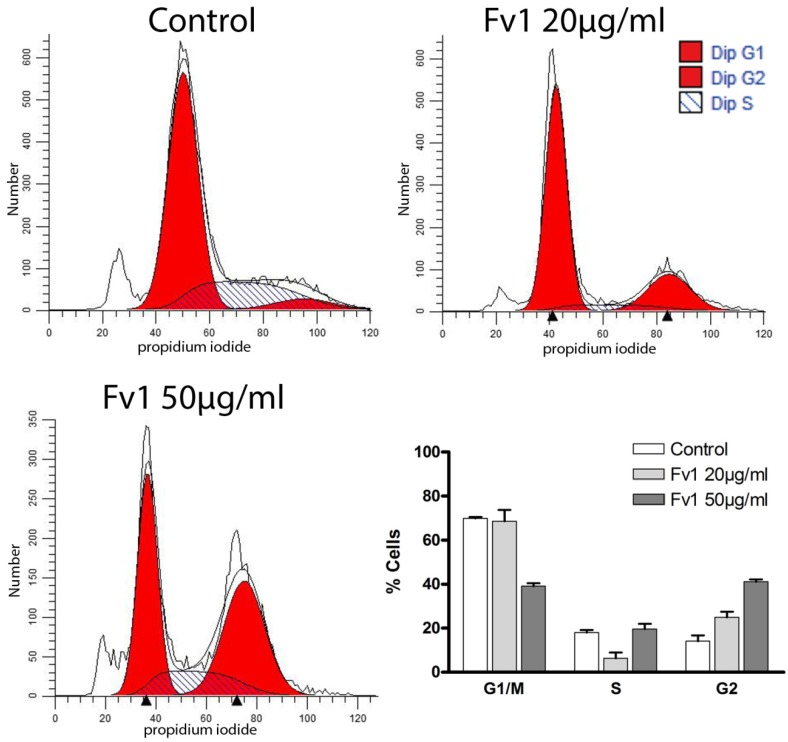
Fv1 induces a cell cycle arrest in pancreatic cancer cells. Colo357 cells were treated with Fv1 24 h after seeding and detached after 24 h of treatment. The detached cells were stained with hypotonic propidium iodide staining and measured by flow cytometry. The data were analyzed with Modfit and presented as percentage of cells for each cell cycle phase. One representative of two replicates is shown. See Supplementary Figures S11–S13 for a 4 h time point and the corresponding experiments with the cell line Panc89. The sub-G1 peak was not considered, because only cells with a cell-like FSC and SSC were taken into account.

To confirm this hypothesis, we examined changes in cell cycle by propidium iodide staining. Treatment with Fv1 led to an elevated number of cells in G2 phase and a decrease of cells in G1 and S phase (Figure 4). This suggests a cell cycle inhibition in the G2 phase, which was also observed in Panc89 cells (see Supplementary Figures S12 and S13).

**Figure 5:**
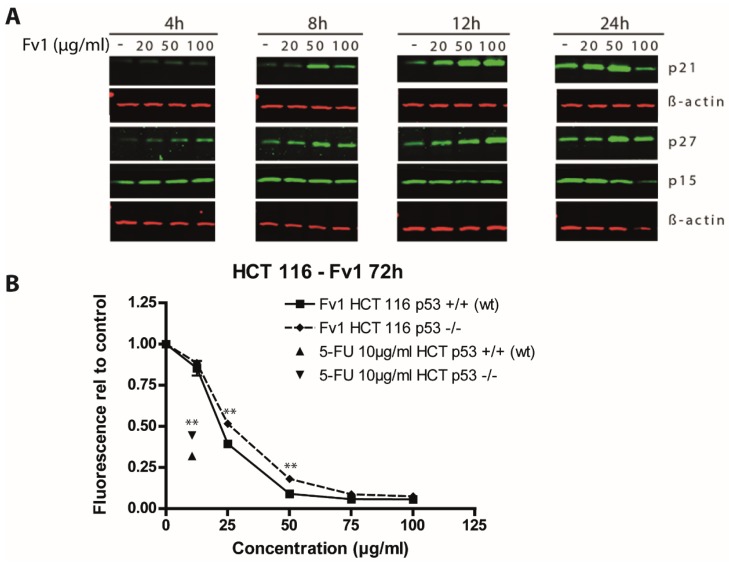
Effect of Fv1 on cell cycle regulating proteins. (**A**) Panc89 cells were grown for 24 h in 6 well plates and treated with different concentrations of Fv1. At the indicated time points, cells were lysed and lysates were analyzed by Western blotting. Proteins were detected using fluorescence-labeled secondary antibodies and an infrared scanner (Odyssey, LICOR); (**B**) HCT 116 cells with wild-type p53 or loss of p53 were treated with different concentrations of Fv1 for 72 h. 5-Fluorouracil at 10 µg/mL was used as control. Viability was measured with AlamarBlue. The experiment was performed in biological triplicates. Significance was calculated using students *t*-test. Differences with a *p*-value <0.001 are indicated with **. One of 3 replicates is shown as representative experiment.

In addition, we analyzed some of the de-regulated genes on the protein level by Western blotting. The cell cycle inhibitors p21 and p27 were induced by Fv1 after 4 to 24 h as shown for the PDAC cell line Panc89 (Figure 5A). These findings support our hypothesis of a cell cycle affecting mechanism induced by Fv1. Analysis of cyclin D3, cyclin E1, p15 and p16 showed no differences compared to β-Actin or α-Tubulin as reference controls (see Supplementary Figures S2 and S3).

The upstream regulator and one of the most important cell cycle regulators is p53. We analyzed its role in response to Fv1 using a p53 knockout model generated with the cell line HCT 116. The p53-inducing drug 5-Fluorouracil was used as control. The effect of Fv1 was significantly lower in the p53-lacking (−/−) cells than in the p53 wild-type variant (Figure 5B).

### 2.3. Impact of Caspase Activity and Autophagy

**Figure 6:**
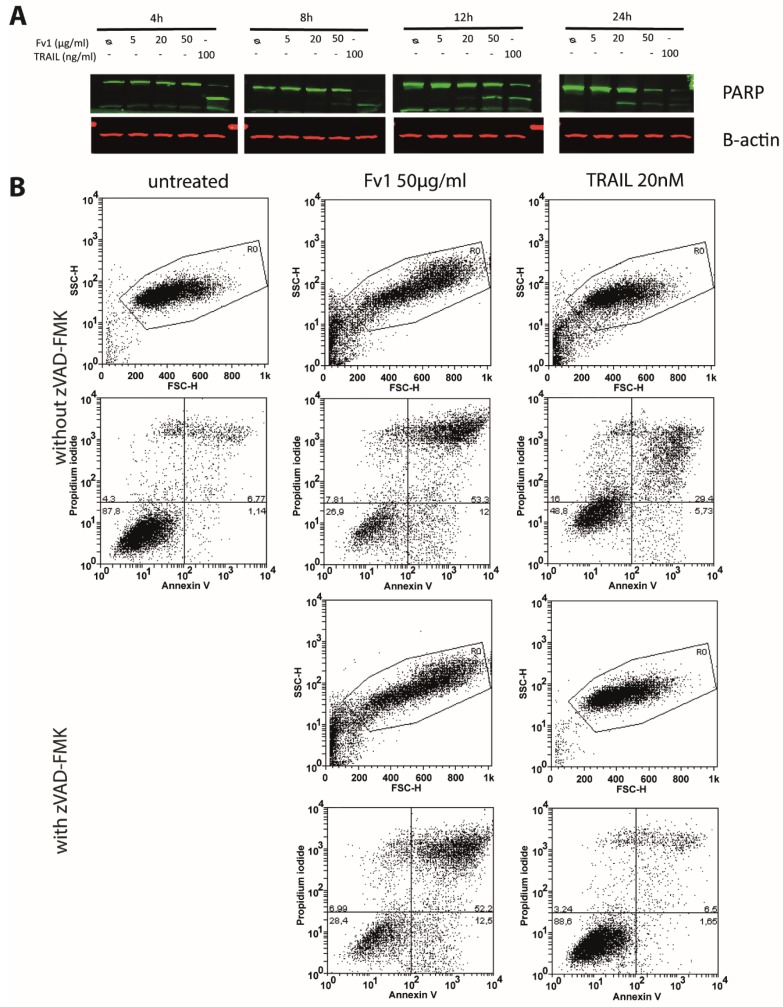
Fv1 induces cleavage of Poly ADP ribose polymerase 1 (PARP), but cell death is not blocked by the caspase inhibitor zVAD-fmk. (**A**) 24 h after seeding, Panc89 cells were treated with Fv1. At the indicated time points, whole cell lysates were analyzed by Western blotting for the cleavage of PARP; (**B**) Panc89 cells were treated with Fv1 as described above, but in combination with zVAD-fmk for 24 h. Afterwards, cells were stained with Annexin V and propidium iodide and measured by flow cytometry. Viability of Panc89 cells treated with Fv1 and zVAD-fmk was observed by AlamarBlue staining after 72 h of treatment (see Supplementary Figure S4).

Analysis of PARP via Western blot showed a cleavage when high concentrations of Fv1 were applied (Figure 6A). Analysis of cell death via co-staining with Annexin V and propidium iodide showed no clear differentiation between apoptosis and necrosis, but a general dose-dependent cell death after 24 h of treatment (Figure 6B). Treated cells were larger in size (FSC) and more granulated (SSC) than normal cells. While the caspase inhibitor zVAD-fmk inhibited the cytotoxic effect of the prototypical member of the death ligand family, TRAIL, it had no impact on the Fv1 activity (Figure 6B). In addition, zVAD-fmk did not inhibit the anti-proliferative effect as observed by AlamarBlue assays (see Supplementary Figure S4).

Cell death is often mediated through the mitochondrial pathway. The production of reactive oxygen species (ROS) and mitochondrial swelling were not induced when Colo357 cells were treated with Fv1 for up to 60 min. Moreover, the membrane potential of isolated rat liver mitochondria was not influenced by Fv1 treatment (Supplementary Figures S5–S8).

**Figure 7:**
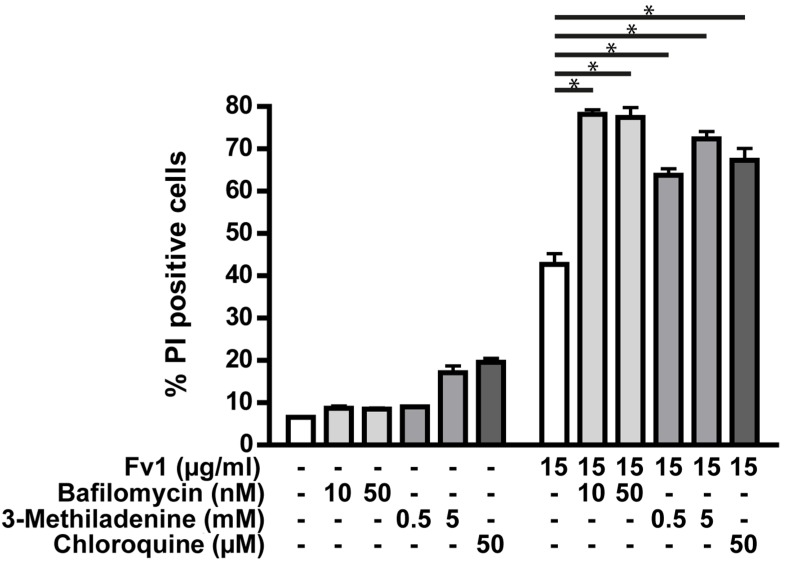
Autophagy inhibitors increase the effect of Fv1. Colo357 cells were prestimulated 23 h after seeding with chloroquine (50 µM), bafilomycin (10 nM, 50 nM) or 3-methyladenine (0.5 mM, 5 mM). One hour later, they were treated with Fv1 (15 µg/mL). After 24 h of Fv1 treatment, cells were detached with accutase, stained with propidium iodide and measured by FACS. Single inhibitor treatments were subtracted from the combination treatments before calculating significances with *t*-tests. *n* = 2.

Another mechanism possibly involved in cell death is autophagy. The dependency of Fv1 on autophagy was analyzed using three different inhibitors of autophagy pathways (Figure 7). Interestingly, the effect of Fv1 was increased when autophagy was blocked by each of the three inhibitors.

### 2.4. Analysis of the Fv1 Effect on Non-Dividing Cells

**Figure 8:**
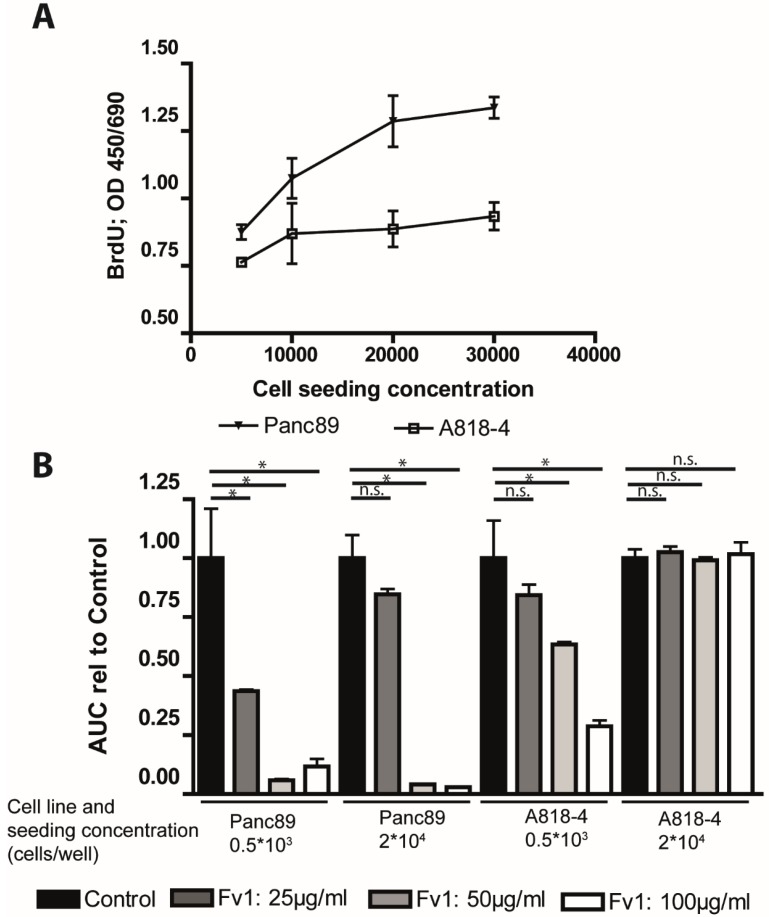
Fv1 preferentially inhibits proliferating cells. (**A**) The PDAC cell lines A818-4 and Panc89 were seeded at different densities. After 24 h, the cells were incubated with BrdU for 4 h. Subsequently, a BrdU ELISA was performed, and the BrdU incorporation was analyzed by measuring the absorbance at 450 nm. The slopes of the curves are significantly different (*p* = 0.000315); (**B**) A818-4 and Panc89 cells were seeded in a CIM 16 plates. After 24 h, they were incubated with different concentrations of Fv1. For 72 h, the cell impedance was monitored in intervals of 15 min by the Roche XCelligence RTCA system. The cell number is represented by the area under the curve (AUC) which is presented as bar chart. *n* = 2. The experiment was validated with an AlamarBlue viability assay which shows the endpoint situation after 72 h of treatment (Supplementary Figure S9). *n* = 2.

As many chemotherapeutics depend on cell proliferation, we analyzed the effect of Fv1 on non-proliferating cells. To do so, we used the PDAC A818-4 cell line model [18]. These cells can be transferred into a quiescent state by contact inhibition (previous unpublished data of our group). Panc89 cells were used as a negative control, representing a common type of cancer cell line that is not influenced by contact inhibition. Accordingly, Panc89 cells incorporated more BrdU when they were seeded at higher numbers, while the incorporation of BrdU in A818-4 cells did not increase with the higher seeding density (Figure 8A).

To analyze the effect of Fv1 on these non-proliferating cells, we monitored them in real-time by the XCelligence RTCA system. Fv1 dose-dependently decreased the cell numbers of A818-4 cells when the seeding density was low. In contrast, Fv1 had no effect upon growth-arrested cells (Figure 8B). Cell numbers are represented as area under the curve (AUC). 

Panc89 cells, on the other hand, were inhibited at both seeding densities (Figure 8B). This finding was confirmed with a viability endpoint measurement after 72 h (Supplementary Figure S9).

**Figure 9:**
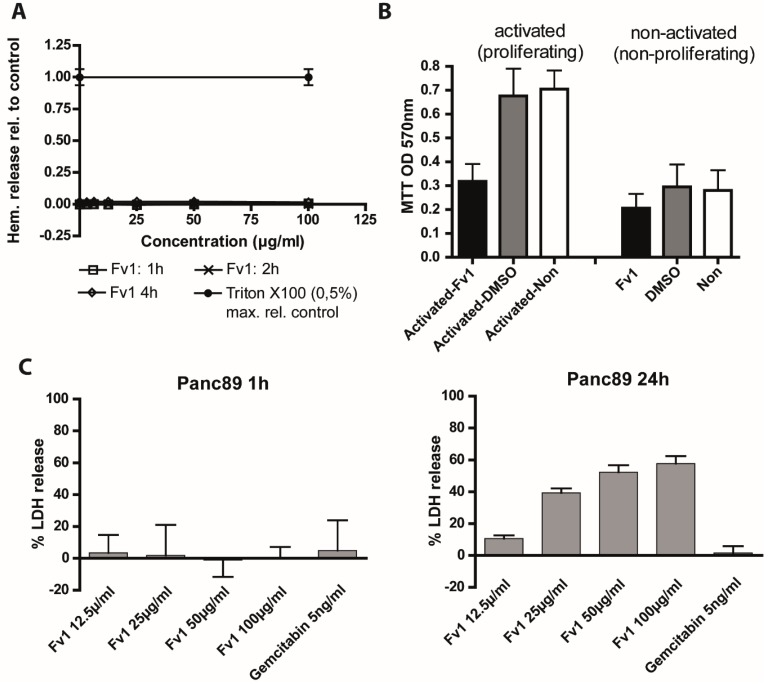

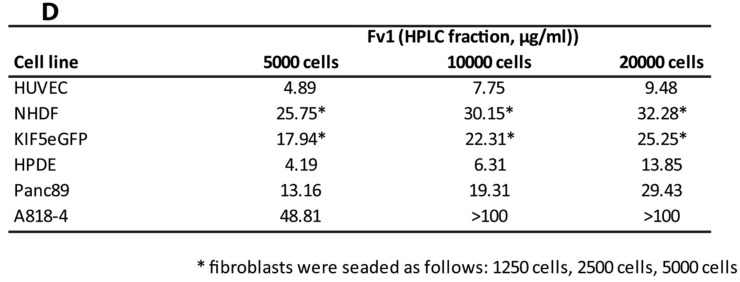
Fv1 treatment does not lead to acute cytotoxicity but effects rapidly dividing non-malignant cells. (**A**) Freshly isolated red blood cells were seeded in 96 well plates and treated with different concentrations of Fv1. Absorbance of hemoglobin was measured in the supernatant at the given time points. 0.5% Triton X100 was used as maximum release control. Raw data are shown in Supplementary Table S2. Four technical replicates are shown; (**B**) Peripheral blood mononuclear cells (PBMCs) were isolated from freshly drawn human blood and activated. Resting or activated cells were treated with Fv1 or DMSO as solvent control. After 72 h, the viability was measured with an MTT assay. Data show raw OD-values. Data are presented as mean values ± standard deviation (SD) from three independent experiments; (**C**) For measurement of acute cytotoxicity, lactate dehydrogenase (LDH) release was measured in Panc89 cells after 1 and 24 h. Experiment performed at least twice with 4 technical replicates each; (**D**) Three non-malignant (HUVEC, NHDF, KIF5eGFP, HPDE) and two PDAC cell lines (Panc89, A818-4) were seeded at different densities. After 24 h, they were treated with Fv1 for 72 h. Dose response curves and EC50 values were calculated using nonlinear regression (curve fit) with Prism (GraphPad). Experiments were performed at least twice with 4 technical replicates each.

For toxicity analysis against sensitive non-malignant body cells, we tested the hemoglobin release of fresh red blood cells derived from healthy donors (Figure 9A). We did not see hemolysis in Fv1-treated red blood cells after 1–4 h of treatment, indicating that Fv1 does not destroy terminally differentiated, non-dividing cells (Supplementary Table S2). Quiescent peripheral blood mononuclear cells (PBMC) were also not affected, while activated proliferating PBMCs showed a reduced viability after 24 h of Fv1 treatment (Figure 9B). 

To characterize the anti-proliferative activity in more detail, we measured acute cytotoxicity by a lactatdehydrogenase (LDH) release assay (Figure 9D). Cancer cell lines did not release LDH after 1 h of treatment with Fv1, but after 24 h we detected a dose-dependent high LDH release (Figure 9C). This indicates a cytotoxic effect of Fv1 after 24 h, but argues against acute cytotoxicity.

To test whether inhibition of proliferation was restricted to cancer cell lines, we tested the effects of Fv1 on different non-malignant cell lines and primary cells (human pancreatic ductal epithelial/HPDE cells, the fibroblast cell lines KIF5 and NHDF and freshly isolated endothelial HUVEC cells). Since these cells were rapidly dividing, we observed similar anti-proliferative effects as in cancer cell lines (Figure 9D).

## 3. Discussion

In this paper, we describe the anticancer effects of a Baltic brown seaweed extract. Fv1 inhibits proliferating cells and does not affect resting cells. This effect is comparable to clinically used chemotherapeutic drugs. Our findings show that the cell line A818-4 was only inhibited by Fv1 when proliferating. In other proliferating cells like Panc89 or Colo357, Fv1 led to an S/G2-phase arrest and to an increase of the cell cycle inhibitors p21, p27 and p57. These findings were supported by the gene expression experiments. Large scale array experiments showed particularly regulation of genes involved in cell cycle regulation. Cell cycle inhibitors were upregulated after Fv1 treatment whereas the expression of downstream targets was decreased. Normally, this enforced form of cell cycle inhibition leads to apoptosis, necroptosis or other forms of programmed cell death [19,20]. Analyses of apoptosis via PARP cleavage showed, that in later stages of Fv1 treatment, a PARP-cleavage occurred, so eventually the cells undergo apoptosis. This hypothesis was supported by Annexin V/PI staining where events of early and late apoptosis/necrosis were observed. However, the cell death was not blocked by the caspase inhibitor zVAD-fmk. Therefore, we assume that it might only be a secondary effect of Fv1 treatment. In addition, Fv1 did not cause mitochondrial swelling or destruction, thus Fv1 did not induce cell death by the mitochondrial pathways. Interestingly, Fv1 seems to be counteracted by autophagy mechanisms. Studies show that autophagy is not only involved in cell death [21,22]. Autophagy pathways also provide a rescue mechanism by stabilizing the cell metabolism [22]. A connection between cell cycle or DNA damage and autophagy is discussed in literature, but not fully understood, yet [23,24,25]. Further *in vivo* experiments with Fv1 could include the combination with inhibitors of autophagy.

Interestingly, some of the described outcomes in the various test systems occur also when cells were treated with Gemcitabine. Gemcitabine is often described to induce apoptosis [26,27] but there is evidence that this could only be a side effect. Studies suggest that the effect of Gemcitabine alone is not influenced by inhibition of caspases [27,28]. This means, that Gemcitabine, Fv1 and other chemotherapeutics induce cell death, but the apoptotic pathway involving caspase activation and PARP cleavage only plays a secondary role.

This supports the hypothesis that Fv1 is a strong inhibitor of the cell cycle like, e.g., Gemcitabine. Indeed, Fv1 led to a cell cycle arrest in G2 phase. This is in line with the microscopic observation that many cells entered the process of mitosis and duplicated their nuclei, but did not finish cytokinesis. The question is how this cell cycle arrest and cell death is mediated. The up-regulated proteins p21, p27 and p57 are all under the control of p53 [19]. Our experiment with p53 knockout cells showed, that there is an influence of p53 on the Fv1 effect. The difference we observed between p53 wild-type and deficient cells was comparable to the one observed for 5-FU. For this DNA-damaging chemotherapeutic agent, the p53 dependency has been described in literature [29]. However, in our other studied cancer cell lines except HCT116 and Colo357, p53 is mutated [18,30]. This is interesting, as Colo357 cells were more resistant to Fv1 than other PDAC cell lines. A connection might be the tumor protein 53 inducible protein 1 (TP53INP1). TP53INP1 is strongly induced by Fv1 on the gene expression level and it is able to play an autonomous role in cell cycle regulation and mediates several of its functions independently of p53 [31]. Another player might be CDC20. This gene was down-regulated by Fv1. It plays an important role in the spindle checkpoint. Eichhorn *et al*. showed that knockdown of CDC20 in HeLa cells induced mitotic arrest, similar to our observations [32]. Moreover, in Western blots, we observed a strong decrease of tubulin at later time points and higher Fv1 concentrations (data shown in Supplementary Figures S2 and S3). This might lead to a destruction of the spindle apparatus and activation of the spindle checkpoint.

Combination experiments with chemotherapeutic drugs indicated that extracts of *Fucus vesiculosus* share completely additive effects with gemcitabine, paclitaxel and cisplatin (Supplementary Figure S10). They neither inhibited nor synergistically enhanced the effects of the other drug. These findings support the assumption that a combination of chemotherapeutic drugs and Fv1 may be effective *in vivo*.

Fv1 is a potent compound with strong anti-cancer activity as shown here under *in vitro* conditions. Subsequently, *in vivo* experiments in mice would be necessary to confirm the promising *in vitro* data and to assess potential side effects such as hepatotoxicity and nephrotoxicity of Fv1. Other compounds like fucoxanthin also show toxic effects on non-malignant cells in cell culture, but have no toxic effect *in vivo* [7]. Experiments in laboratory rodents would also reveal the uptake and bioavailability of Fv1 when applied to complex organisms. Another way of further application would be the use of Fv1 for targeted therapy of cancer. To do so, Fv1 could be conjugated to cancer-specific target molecules or be wrapped by liposomes that can release their content specifically to cancer cells. However, structure and other chemical characteristics of Fv1 need to be elucidated prior clinical trials. In conclusion, Fv1 is a potent inhibitor of cancer cell growth. It does not induce apoptosis or necroptosis, nor does it induce mitochondrial swelling. However, Fv1 efficiently kills pancreatic cancer cells by inducing a cell cycle arrest by the induction of cell cycle inhibitors involving TP53INP1. 

## 4. Material and Methods

### 4.1. Algae Extraction and Fractioning

Freshly frozen parts of the central thallus of the alga *Fucus vesiculosus*, which were collected at spring time from coastal areas of Bülk, Kiel Fjord, Western Baltic Sea, were thawed and extracted with acetone at the rate of 1:2 (*w/v*). After further processing and drying, the extract was re-dissolved in dimethyl sulfoxide (DMSO) as preparation for normal phase high performance liquid chromatography (HPLC) on a Pharmprep60CC SiO_2_ column, followed by reversed-phase HPLC on an Amberlite^®^ XAD7HP column. 67 fractions were generated, dried and dissolved in DMSO D6. 

Fv1 is represented by a pool of three successively eluted fractions within the hydrophilic sector of the applied gradient. According to their similar and most efficient anti-proliferative activity in the sequence of seven tested neighboring fractions and according to their characteristic ^1^H-NMR-profile showing four signals each at 5.82, 6.63, 7.89 and 8.98 ppm, the three nearby fractions were pooled to Fv1 in a concentration of 42 mg/mL in DMSO and stored at −60 °C for subsequent testing.

### 4.2. Cell Lines, Cell Culture Maintenance and General Experiment Procedure

PancTu1 cells were a kind gift of Dr. M. von Bülow (Mainz, Germany), Panc89 cells of Dr. T. Okabe (Tokyo, Japan), and Colo357 cells of Dr. R. Morgan (Denver, CO, USA) [33]. Panc1 cells were obtained from ATCC (LGC Standards, Wesel, Germany). These four pancreatic cancer cell lines were chosen in order to not depend too much on the particular genetic outfit of the cell lines. Most experiments have been done with 2 or more of the cell lines and representative experiments are shown. The E6/E7-HPV16-immortalized human pancreatic ductal epithelial cell line HPDE was a gift of Dr. M. Tsao (MD, FRCPC, University Health Network, Toronto, ON, Canada) [34,35]. NHDF cells were purchased from Promocell (Heidelberg, Germany). Cell culture maintenance and experiments were performed in an incubator at 37 °C with a 5% CO_2_ atmosphere and 5% humidity. HPDE cells were routinely cultured in HPDE medium (RPMI 1640 medium (Life Technologies, Darmstadt, Germany) supplemented with 10% FBS (PAN Biotech, Aidenbach, Germany), 1% Glutamax (Life Technologies, Darmstadt, Germany) and mixed immediately before use 1:1 with keratinocyte medium SFM (Life Technologies, Darmstadt, Germany) supplemented with 0.025% bovine pituitary extract/2.5 µg/L epidermal growth factor (Life Technologies, Darmstadt, Germany)). NHDF cells were cultivated in fibroblast growth medium supplemented with supplement pack 2 (Promocell, Heidelberg, Germany). All other cell lines were cultured in RPMI 1640 medium (Life Technologies, Darmstadt, Germany) supplemented with 10% FBS (PAN Biotech, Aidenbach, Germany), 1% Glutamax and 1% sodium pyruvate ( both Life Technologies, Darmstadt, Germany). Red blood cells were isolated directly from fresh blood from healthy donors by centrifugation and maintained in PBS. Peripheral blood mononuclear cells (PBMC) were isolated and activated with a T cell activation/expansion kit (130-091-441, Miltenyi Biotec, Bergisch-Gladbach, Germany).

### 4.3. Proliferation and Viability Experiments

For proliferation experiments, cells were seeded in 96 well plates (5 × 10^3^ cells per well if not noted otherwise) and treated after 24 h with serial dilutions of extracts premixed in cell culture medium. The algae extract stocks (42 mg/mL in DMSO) were thawed directly before use. Serial dilutions were done in DMSO to ensure a constant DMSO concentration of 0.15% in cell culture medium for each treatment. A DMSO concentration of 0.15% in cell culture medium was used as solvent control. TRAIL (PeproTech, Hamburg, Germany) was dissolved in PBS (0.5 µg/µL) and diluted in cell culture medium in a concentration of 200 ng/mL. zVAD-fmk (Promega, Mannheim, Germany) was dissolved in DMSO and diluted in cell culture medium using a concentration of 20 µM. After the indicated time points, the proliferation tests were performed as described below. Dose response curves were produced and EC50 values calculated with Prism (GraphPad).

### 4.4. Alamar Blue

Ten microliters of AlamarBlue (Invitrogen, Carlsbad, CA, USA) reagent was added to each 96 well containing cells in 100 µL of medium and incubated for 4 h. Fluorescence of AlamarBlue was measured using an excitation wavelength of 545 nm and an emission wavelength of 595 nm (Tecan Spectrafluor Plus, Tecan, Maennedorf, Switzerland). 

### 4.5. BrdU ELISA

For A818-4 cells, a BrdU ELISA was used to determine the optimal cell seeding concentration for contact-inhibited cell growth. Experiments were performed as described before and for the last 5 h, the BrdU labelling reagent was added to the cell culture at a final concentration of 10 µM. The following BrdU-staining was performed according to the manual (Roche, Cat. No. 11647229001). The BrdU-reaction was stopped with 25 µL 1 M H_2_SO_4_. Absorbance was measured using the Tecan Sunrise Reader (Tecan, Maennedorf, Germany) with a wavelength of 450 nm (Reference: 600 nm).

### 4.6. XCelligence Proliferation Measurement

Cells were seeded in XCelligence CIM-plate 16 (AceaBio, San Diego, CA, USA) according to the manufacturer’s instructions in 10% FCS containing RPMI medium. After 24 h, cells were treated as described in the general experiment section for 96 well plates. Proliferation activity was measured every 15 min for 72 h in a RTCA DP system (AceaBio, San Diego, CA, USA).

### 4.7. Cytotoxicity LDH-Release

LDH-release assays were performed using RPMI medium with 1% FCS, 0.5% GlutaMAX and 0.5% sodium-pyruvate for treatment, because these substances can interfere with the assay reagents. 1 × 10^4^ cells per well were seeded in 200 µL cell culture medium. After 24 h attachment, the cells were treated with extracts as described above. For each treatment, half of the wells were also treated with Triton-X100 (1% final concentration in medium) as 100% control. After 1 or 24 h, LDH release was tested according to the manual (Takara Bio, Saint-Germain-en-Laye, France) and absorbance was measured in a Sunrise plate reader at 492 nm with a reference wavelength of 650 nm (Tecan, Maennedorf, Switzerland).

### 4.8. Gene Expression Analysis

For Agilent gene chip expression analysis, 1.07 × 10^6^ cells were grown in 12 mL cell culture medium in T75 flasks for 24 h before treatment. Cells were treated with 30 µg/mL Fv1 or 0.15% DMSO as control. After an incubation time of 6 or 24 h, the supernatant was removed, the cells were washed with PBS and detached with Accutase and frozen at −80 °C. Then the RNA was extracted with the RNeasy Plus Mini Kit (Qiagen, Hilde, Germany), according to the manual. Quantity of the RNA was measured using NanoDrop (PeqLab). Experiments were performed in three independent replicates. RNA quality was controlled using a BioAnalyzer. Gene expression was measured using the “Sureprint G3 Human GE 8 × 60K” (Agilent, Santa Clara, CA, USA) array which was processed according to the manufacturer’s instructions. For analysis, only genes were included that showed a *p*-value < 0.05 and a log fold change (lfc) >1 or <−1 and that were changed on at least three pancreatic cancer cell lines (Panc1, Panc89, PancTU1). The experiments were performed as three biological replicates with each cell line.

### 4.9. Preparation of Cell Lysates and Western Blotting

Cells were lysed with RIPA buffer and ultrasound. Protein concentration was measured with DC reagent (Bio-Rad Laboratories, Munich, Germany). Equal amounts of protein were loaded on a 4%–20% tris-glycine gel (Novex, Life Technologies, Carlsbad, CA, USA) and separated by SDS-PAGE. Proteins were transferred by semi dry blot on a PVDF membrane (Immobilon-FL; Millipore/Merck, Darmstadt, Germany). For 2nd antibody, goat-anti-rabbit-IRDye800CW and goat-anti-mouse-IRDye680 (LI-COR, Bad Homburg, Germany) were used. Blots were dried and scanned using an Odyssey infrared imager (LI-COR, Bad Homburg, Germany). Used antibodies: β-Actin (CS, A5441), α-Tubulin (Epitomics, 1878-1), PARP (CS, 9542S), p21 (CS, 2946), p27 (CS, 2552), p15 (CS, 4822).

### 4.10. FACS Cell Cycle Profiling—Annexin V/PI Staining

For cell cycle profiling, experiments were performed as described above. Staining was performed in v-bottom shaped 96 well plates using a hypotonic propidium iodide (PI) staining solution (0.1% sodium citrate, 0.1% TritonX-100, 50 µg/mL PI, 5 µg/mL EDTA) [36]. Stained cells were measured immediately after staining using a FACS-Calibur (BD Biosciences, Heidelberg, Germany). Annexin V/PI staining was performed according to the manufacturer’s instructions (Miltenyi Biotec; 130-092-052). Immediately after adding the PI solution, the cells were measured in FACS-Calibur (BD Biosciences, Heidelberg, Germany).

### 4.11. FACS PI Staining and Inhibitor Screening

One hundred thousand (1 × 10^5^) Colo357 cells were seeded in 12 well cell culture plates in 0.5 mL cell culture medium. After 23 h they were pretreated with Bafilomycin (10 nM, 50 nM), Chloroquine (50 µM), 3-Methyadenine (0.5 mM, 5 mM) or cell culture medium (control). One hour later, 15 µg/mL Fv1 or cell culture medium (control) was added. After 24 h, the cells were detached with Accutase (PAA), stained with propidium iodide and measured with a FACS-Calibur (BD, Heidelberg, Germany). The percentage of PI positive cells was determined for each treatment.

### 4.12. Statistical Analysis

Statistics were performed with Prism (GraphPad) and Excel (Microsoft) unless indicated otherwise. Significance was tested using students *t*-test. Differences with *p*-values <0.05 (*), <0.01 (**) or <0.001 (***) were estimated as significant and indicated with asterisk.

## Supplementary Materials

**Table S1.** Inhibition of cell viability by Fv1 in different cancer cell lines.
**Figure S1.** Genes involved in the cell cycle regulation pathway are regulated by Fv1.
**Figure S2.** Fv1 does not influence several proteins involved in cell cycle regulation.
**Figure S3.** Fv1 does not influence several proteins involved in cell cycle regulation and cell death.
**Figure S4.** Supplement to Figure 6. The effect of Fv1 on viability is not caspase dependent.
**Figure S5.** Mitosox ROS production of Colo357 cells treated with Fv1.
**Figure S6.** Mitosox ROS production of Colo357 cells treated with Antimycin as positive control.
**Figure S7.** TMRM membrane potential of Colo357 cells treated with Fv1.
**Figure S8.** TMRM membrane potential of Colo357 cells treated with FCCP as positive control.
**Figure S9.** Supplement to Figure 8. Fv1 preferentially inhibits proliferating cells.
**Table S2.** Fv1 does not induce hemolysis in fresh red blood cells.
**Figure S10.** Acetonic algae extracts show additive effects in combinations with different chemotherapeutics.
**Figure S11.** Cell cycle profile of Colo357 cells 4 h after treatment with Fv1.
**Figure S12.** Cell cycle profile of Panc89 cells 4 h after treatment with Fv1.
**Figure S13.** Cell cycle profile of Panc89 cells 24 h after treatment with Fv1.
**Multimedia file S1.** Life cell imaging Control.
**Multimedia file S2.** Life cell imaging Fv1.

## References

[1] SEER Cancer Statistics Review, 1975–2011. http://seer.cancer.gov/csr/1975_2011/.

[2] Ferlay J., Soerjomataram I., Dikshit R., Eser S., Mathers C., Rebelo M., Parkin D.M., Forma D., Bray F. (2015). Cancer incidence and mortality worldwide: Sources, methods and major patterns in GLOBOCAN 2012: Globocan 2012. Int. J. Cancer.

[3] Rahib L., Smith B.D., Aizenberg R., Rosenzweig A.B., Fleshman J.M., Matrisian L.M. (2014). Projecting cancer incidence and deaths to 2030: The unexpected burden of thyroid, liver, and pancreas cancers in the United States. Cancer Res..

[4] Ale M.T., Maruyama H., Tamauchi H., Mikkelsen J.D., Meyer A.S. (2011). Fucose-Containing Sulfated Polysaccharides from Brown Seaweeds iNhibit Proliferation of Melanoma Cells and Induce Apoptosis by Activation of Caspase-3 *in vitro*. Mar. Drugs.

[5] Kumar S.R., Hosokawa M., Miyashita K. (2013). Fucoxanthin: A Marine Carotenoid Exerting Anti-Cancer Effects by Affecting Multiple Mechanisms. Mar. Drugs.

[6] Glombitza K.W., Hauperich S., Keusgen M. (1997). Phlorotannins from the brown algae *Cystophora torulosa* and *Sargassum spinuligerum*. Nat. Toxins.

[7] Khotimchenko Y.S. (2010). Antitumor properties of nonstarch polysaccharides: Fucoidans and chitosans. Russ. J. Mar. Biol..

[8] Park H.S., Kim G.-Y., Nam T.-J., Deuk Kim N., Hyun Choi Y. (2011). Antiproliferative Activity of Fucoidan Was Associated with the Induction of Apoptosis and Autophagy in AGS Human Gastric Cancer Cells. J. Food Sci..

[9] Ye G., Lu Q., Zhao W., Du D., Jin L., Liu Y. (2014). Fucoxanthin induces apoptosis in human cervical cancer cell line HeLa via PI3K/Akt pathway. Tumor Biol..

[10] Wang S.K., Li Y., White W.L., Lu J. (2014). Extracts from New Zealand *Undaria pinnatifida* Containing Fucoxanthin as Potential Functional Biomaterials against Cancer *in vitro*. J. Funct. Biomater..

[11] Ale M.T., Maruyama H., Tamauchi H., Mikkelsen J.D., Meyer A.S. (2011). Fucoidan from *Sargassum* sp. and *Fucus vesiculosus* reduces cell viability of lung carcinoma and melanoma cells *in vitro* and activates natural killer cells in mice *in vivo*. Int. J. Biol. Macromol..

[12] Athukorala Y., Ahn G.N., Jee Y.-H., Kim G.-Y., Kim S.-H., Ha J.-H., Ha J.-H., Kang J.S., Lee K.-W., Jeon Y.-J. (2008). Antiproliferative activity of sulfated polysaccharide isolated from an enzymatic digest of *Ecklonia cava* on the U-937 cell line. J. Appl. Phycol..

[13] Peng J., Yuan J.-P., Wu C.-F., Wang J.-H. (2011). Fucoxanthin, a Marine Carotenoid Present in Brown Seaweeds and Diatoms: Metabolism and Bioactivities Relevant to Human Health. Mar. Drugs.

[14] Kang H.S., Chung H.Y., Kim J.Y., Son B.W., Jung H.A., Choi J.S. (2004). Inhibitory phlorotannins from the edible brown alga *Ecklonia stolonifera* on total reactive oxygen species (ROS) generation. Arch. Pharm. Res..

[15] Ahn J.-H., Yang Y.-I., Lee K.-T., Choi J.-H. (2015). Dieckol, isolated from the edible brown algae *Ecklonia cava*, induces apoptosis of ovarian cancer cells and inhibits tumor xenograft growth. J. Cancer Res. Clin. Oncol..

[16] Yoon J.-S., Kasin Yadunandam A., Kim S.-J., Woo H.-C., Kim H.-R., Kim G.-D. (2013). Dieckol, isolated from *Ecklonia stolonifera*, induces apoptosis in human hepatocellular carcinoma Hep3B cells. J. Nat. Med..

[17] Shirayama M., Toth A., Galova M., Nasmyth K. (1999). APC^Cdc20^ promotes exit from mitosis by destroying the anaphase inhibitor Pds1 and cyclin Clb5. Nature.

[18] Sipos B., Möser S., Kalthoff H., Török V., Löhr M., Klöppel G. (2003). A comprehensive characterization of pancreatic ductal carcinoma cell lines: Towards the establishment of an *in vitro* research platform. Virchows Arch..

[19] Weinberg R. (2013). The Biology of Cancer.

[20] Ouyang L., Shi Z., Zhao S., Wang F.-T., Zhou T.-T., Liu B., Bao J.K. (2012). Programmed cell death pathways in cancer: A review of apoptosis, autophagy and programmed necrosis. Cell Prolif..

[21] Madeo F., Zimmermann A., Maiuri M.C., Kroemer G. (2015). Essential role for autophagy in life span extension. J. Clin. Invest..

[22] Czarny P., Pawlowska E., Bialkowska-Warzecha J., Kaarniranta K., Blasiak J. (2015). Autophagy in DNA Damage Response. Int. J. Mol. Sci..

[23] Hanahan D., Weinberg R.A. (2011). Hallmarks of Cancer: The Next Generation. Cell.

[24] Zhang D., Tang B., Xie X., Xiao Y.-F., Yang S.-M., Zhang J.-W. (2015). The Interplay Between DNA Repair and Autophagy in Cancer Therapy. Cancer Biol. Ther..

[25] Wang F., Li H., Yan X.-G., Zhou Z.-W., Yi Z.-G., He Z.-X., Pan X.T., Yang Y.X., Wang Z.Z., Zhang X. (2015). Alisertib induces cell cycle arrest and autophagy and suppresses epithelial-to-mesenchymal transition involving PI3K/Akt/mTOR and sirtuin 1-mediated signaling pathways in human pancreatic cancer cells. Drug Des. Devel. Ther..

[26] Von Hoff D.D., Ervin T., Arena F.P., Chiorean E.G., Infante J., Moore M., Seay T., Tjulandin S.A., Ma W.W., Saleh M.N. (2013). Increased Survival in Pancreatic Cancer with nab-Paclitaxel Plus Gem Citabine. N. Engl. J. Med..

[27] Wei W.-T., Chen H., Wang Z.-H., Ni Z.-L., Liu H.-B., Tong H.-F., Guo H.C., Liu D.L., Lin S.Z. (2012). Enhanced Antitumor Efficacy of Gemcitabine by Evodiamine on Pancreatic Cancer via Regulating PI3K/Akt Pathway. Int. J. Biol. Sci..

[28] Stadel D., Cristofanon S., Abhari B.A., Deshayes K., Zobel K., Vucic D., Debatin K.M., Fulda S. (2011). Requirement of Nuclear Factor κB for Smac Mimetic-Mediated Sensitization of Pancreatic Carcinoma Cells for Gemcitabine-Induced Apoptosis. Neoplasia.

[29] Sui X., Kong N., Wang X., Fang Y., Hu X., Xu Y., Chen W., Wang K., Li D., Jin W. (2014). JNK confers 5-fluorouracil resistance in p53-deficient and mutant p53-expressing colon cancer cells by inducing survival autophagy. Sci. Rep..

[30] Yoon M., Mitrea D.M., Ou L., Kriwacki R.W. (2012). Cell cycle regulation by the intrinsically disordered proteins p21 and p27. Biochem. Soc. Trans..

[31] Seillier M., Peuget S., Dusetti N.J., Carrier A., El-Missiry M.A. (2012). Antioxidant Role of p53 and of Its Target TP53INP1. Antioxidant. Enzyme.

[32] Eichhorn J.M., Sakurikar N., Alford S.E., Chu R., Chambers T.C. (2013). Critical role of anti-apoptotic Bcl-2 protein phosphorylation in mitotic death. Cell Death Dis..

[33] Morgan R.T., Woods L.K., Moore G.E., Quinn L.A., McGavran L., Gordon S.G. (1980). Human cell line (COLO 357) of metastatic pancreatic adenocarcinoma. Int. J. Cancer.

[34] Furukawa T., Duguid W.P., Rosenberg L., Viallet J., Galloway D.A., Tsao M.S. (1996). Long-term culture and immortalization of epithelial cells from normal adult human pancreatic ducts transfected by the E6E7 gene of human papilloma virus 16. Am. J. Pathol..

[35] Ouyang H., Mou L., Luk C., Liu N., Karaskova J., Squire J., Tsao M.-S. (2000). Immortal Human Pancreatic Duct Epithelial Cell Lines with Near Normal Genotype and Phenotype. Am. J. Pathol..

[36] Krishan A. (1975). Rapid flow cytofluorometric analysis of mammalian cell cycle by propidium iodide staining. J. Cell Biol..

