# Supplementary material for: Molecular Mechanisms by Which a Fucus vesiculosus Extract Mediates Cell Cycle Inhibition and Cell Death in Pancreatic Cancer Cells
Source: Mar Drugs. 2015 Jul 20;13(7):4470–91. doi: 10.3390/md13074470 (PMC4515628; doi:10.3390/md13074470)
Supplement: Supplementary file 3 [file marinedrugs-13-04470-s003.docx]

**Supplementary Information**

**Table S1.** Inhibition of cell viability by Fv1 in different cancer cell lines. 5 × 10^3^ cells were seeded in 96 well plates and treated with Fv1 or DMSO as control (0.15%) after 24 h. After 72 h treatment, an AlamarBlue viability assay was performed. Values are presented as % of control. Data are also shown in Figure 1.

| **Concentration (μg/mL)** | **Panc89** | | **PancTU1** | | **Colo357** | | **Panc1** | |
| --- | --- | --- | --- | --- | --- | --- | --- | --- |
|  | **Mean** | **SD** | **Mean** | **SD** | **Mean** | **SD** | **Mean** | **SD** |
| 0 | 100.0 | 1.0 | 100.0 | 2.1 | 100.0 | 1.4 | 100.0 | 3.7 |
| 0.78 | 97.9 | 0.4 | 102.1 | 1.5 | 100.4 | 1.5 | 101.7 | 2.6 |
| 1.56 | 95.6 | 0.5 | 93.3 | 4.3 | 96.9 | 2.4 | 99.5 | 2.8 |
| 3.13 | 94.6 | 1.4 | 91.8 | 6.0 | 96.7 | 0.8 | 97.5 | 3.0 |
| 6.25 | 94.1 | 1.2 | 91.1 | 6.1 | 97.2 | 0.9 | 90.7 | 1.6 |
| 12.5 | 84.5 | 1.2 | 84.4 | 2.6 | 103.0 | 1.5 | 81.1 | 2.0 |
| 15 | 67.4 | 1.3 | 58.6 | 1.2 | 105.0 | 1.6 | 63.9 | 2.2 |
| 20 | 35.2 | 0.9 | 36.8 | 2.0 | 99.6 | 1.6 | 51.7 | 2.4 |
| 25 | 13.8 | 1.0 | 20..9 | 0.9 | 81.0 | 1.7 | 30.1 | 1.3 |
| 50 | 0.0 | 0.1 | 0.0 | 0.6 | 0.00 | 0.8 | 0.0 | 0.9 |


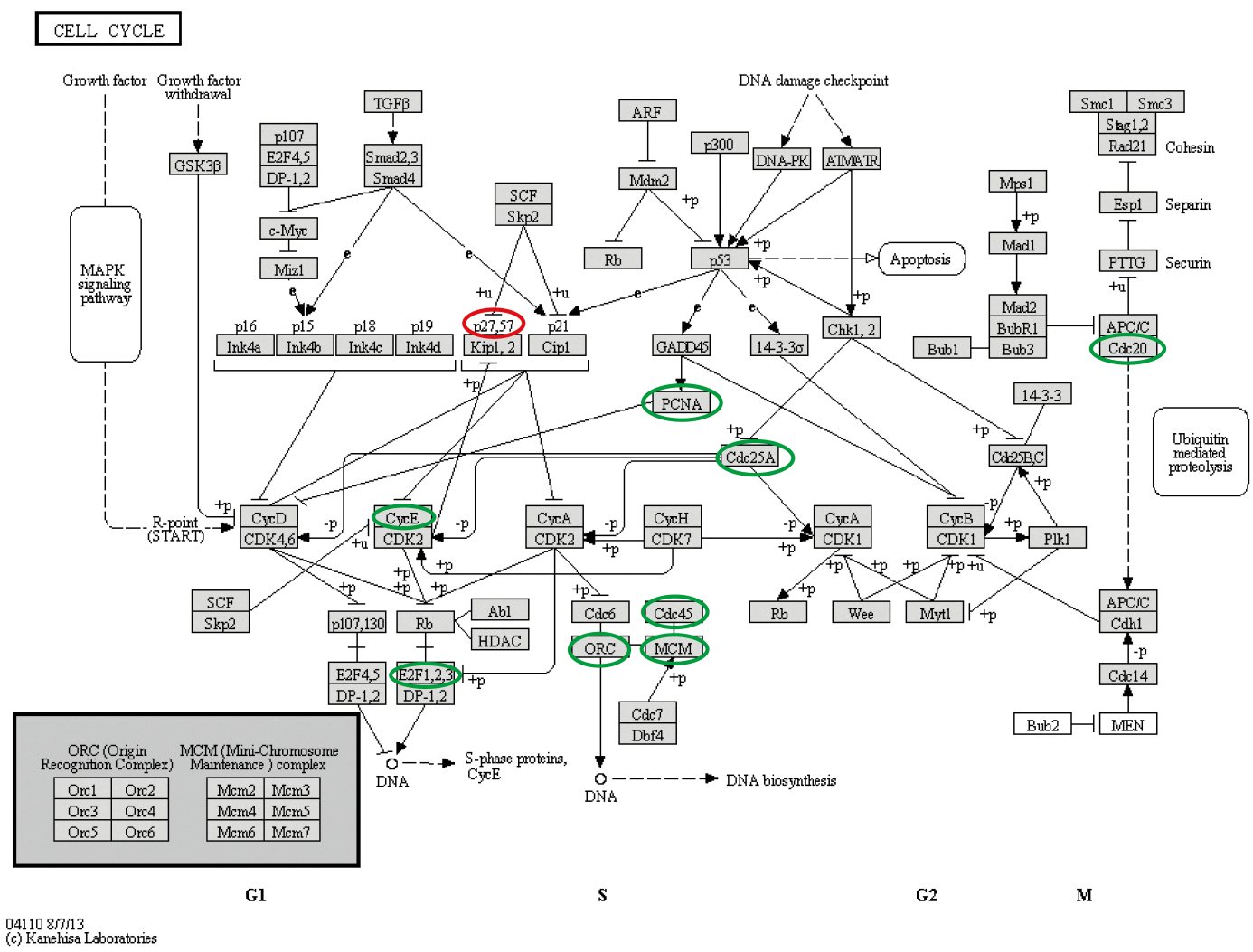


**Figure S1.** Genes involved in cell cycle regulation are regulated by Fv1, analyzed as large scale gene expression screening as described in Table 1: Analyzis was performed with DAVID and KEGG pathways.





**Figure S2.** Fv1 does not influence several proteins involved in cell cycle regulation. Panc89 cells were treated with different concentrations of Fv1 24 h after seeding. At the indicated time points, whole cell lysates were produced and analyzed via Western blot. Readout occurred with fluorophore coupled secondary antibodies and an infrared imager.





**Figure S3.** Fv1 does not influence several proteins involved in cell cycle regulation, proliferation and apoptosis. Colo357 cells were treated with different concentrations of Fv1 24 h after seeding. At the indicated time points, whole cell lysates were produced and analyzed via Western blot. Readout occurred with fluorophore coupled secondary antibodies and an infrared imager.


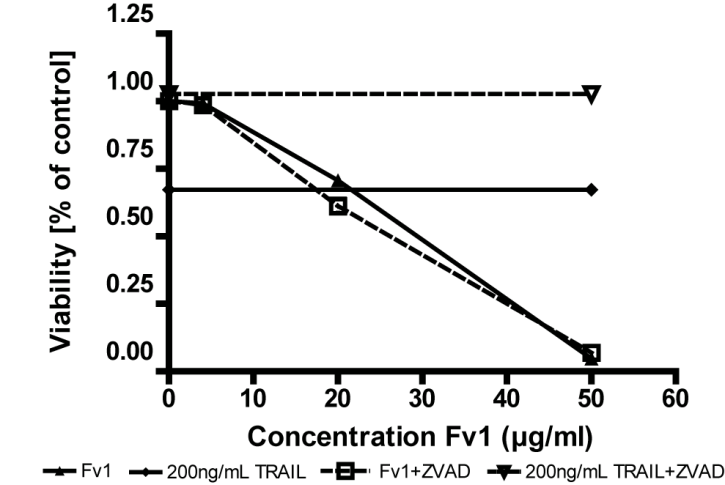


**Figure S4.** Supplement to Figure 6. The effect of Fv1 on viability is not caspase dependent. Panc89 cells were treated 24 h after treatment with different concentrations of Fv1 in combination or without the caspase inhibitor zVAD-fmk. TRAIL was used as control. After 72 h, the viability was measured using AlamarBlue assay. Significance was tested with student *t*-test, assuming a *p*-value < 0.05 as significant.


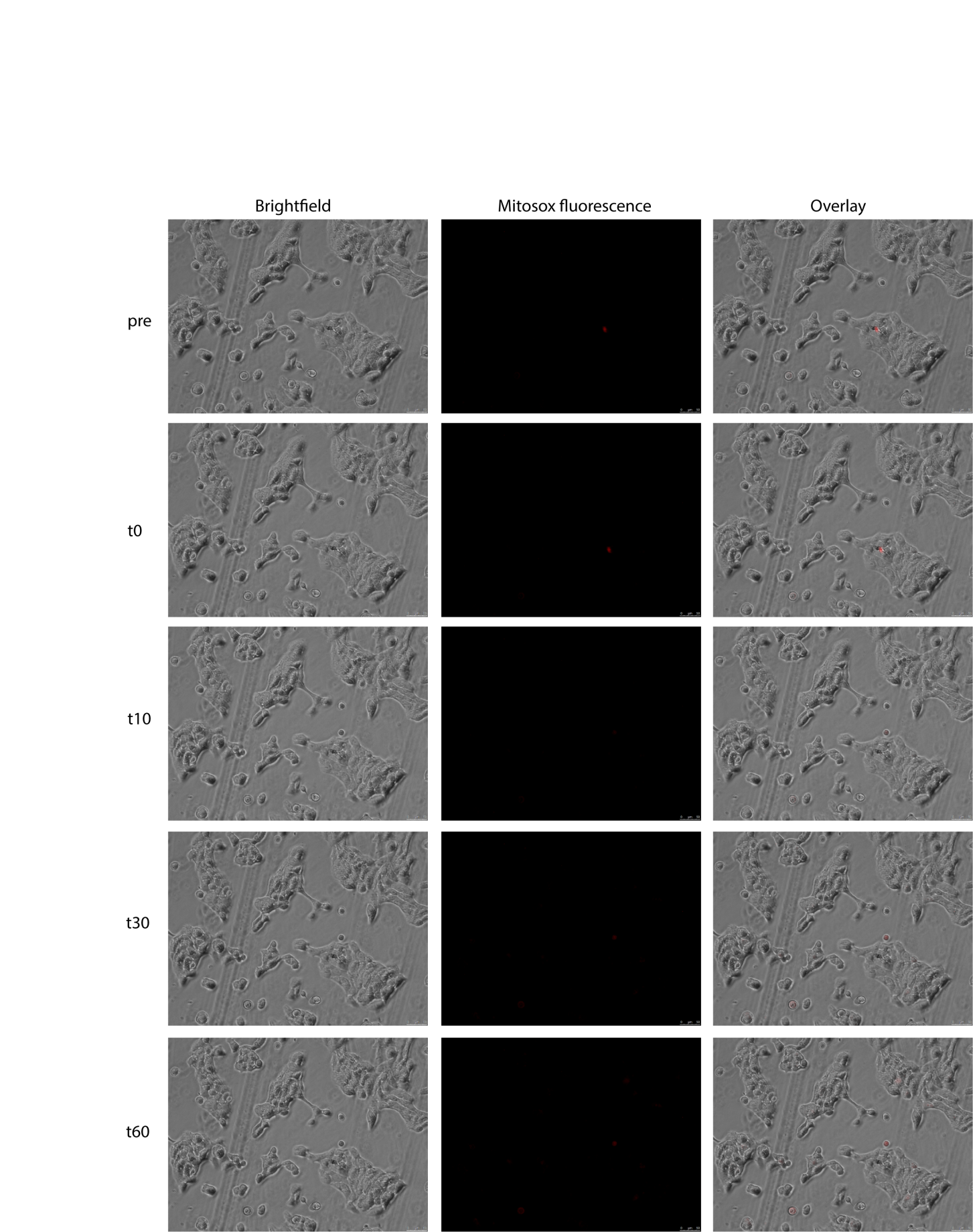


**Figure S5.** Mitosox ROS production of Colo357 cells treated with Fv1. Colo357 cells were treated with 50 µg/mL Fv1. Pictures were taken every 10 min. Representative pictures of indicated timepoints are shown.


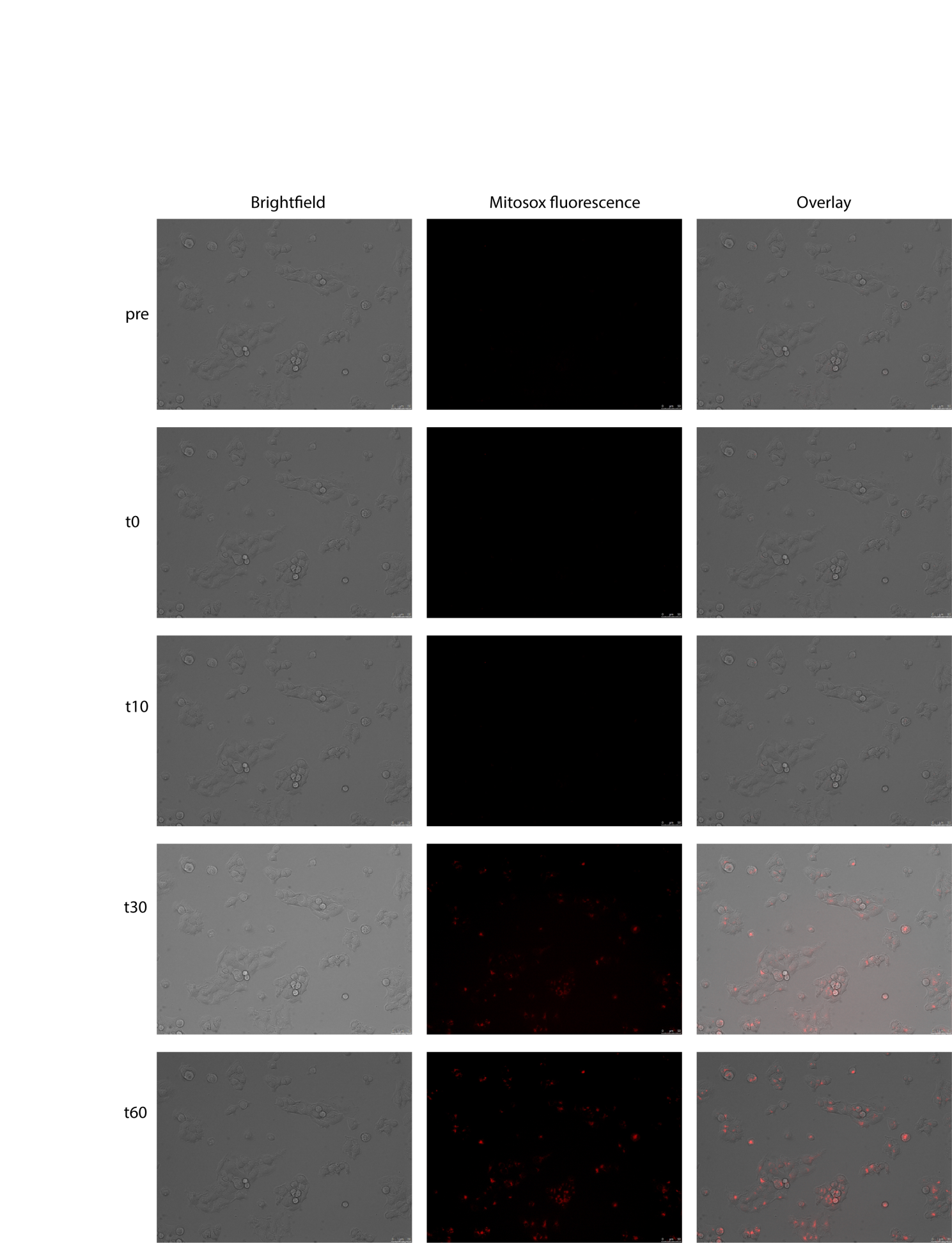


**Figure S6.** Mitosox ROS production of Colo357 cells treated with Antimycin as positive control. Colo357 cells were treated with 4 µg/mL Antimycin. Pictures were taken every 10 min. Representative pictures of indicated timepoints are shown. The fluorescence is increasing over time, indicating an increasing ROS production.


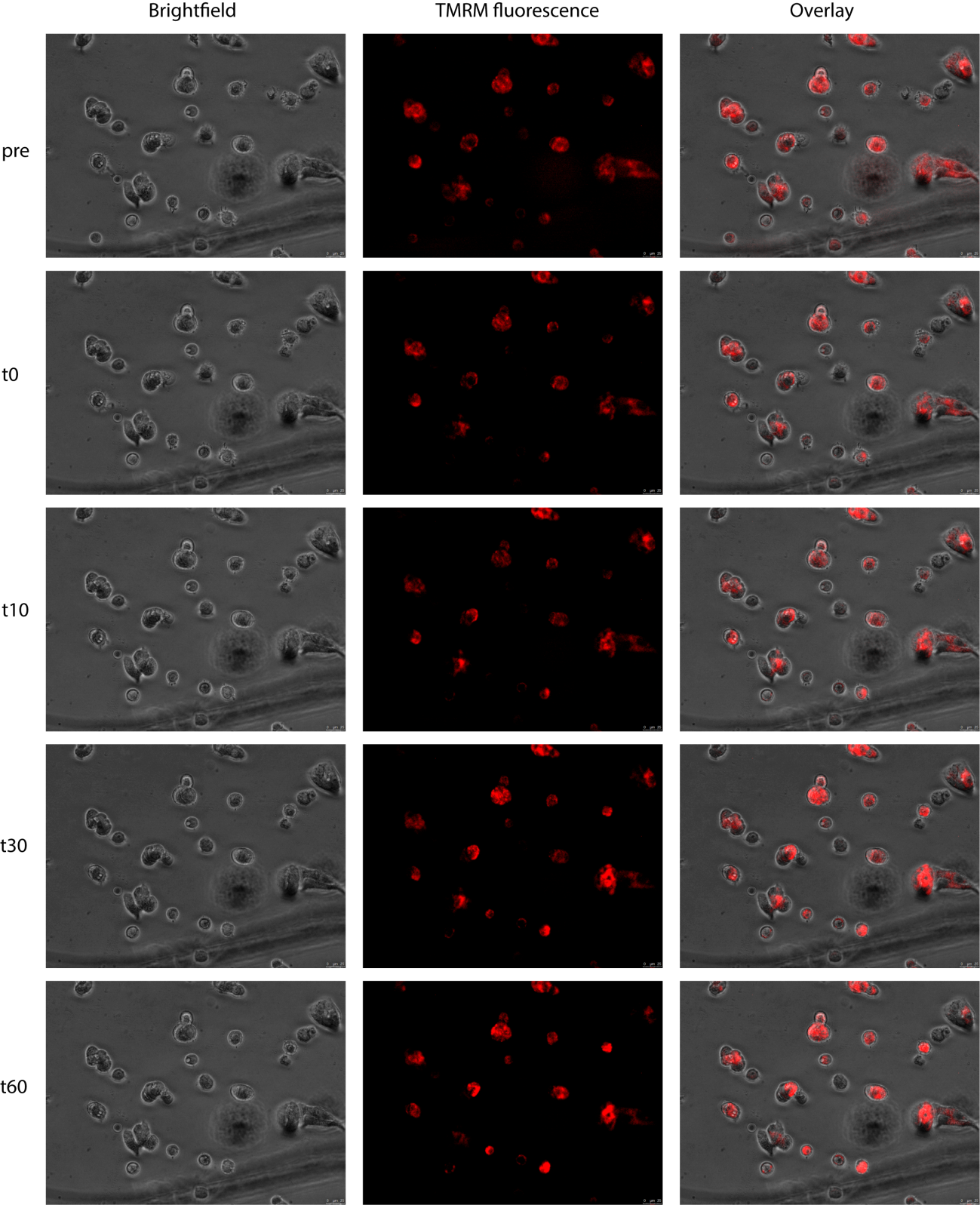


**Figure S7.** TMRM membrane potential of Colo357 cells treated with Fv1. Colo357 cells were treated with 50 µg/mL Fv1. Pictures were taken every 10 min. Representative pictures of indicated timepoints are shown.


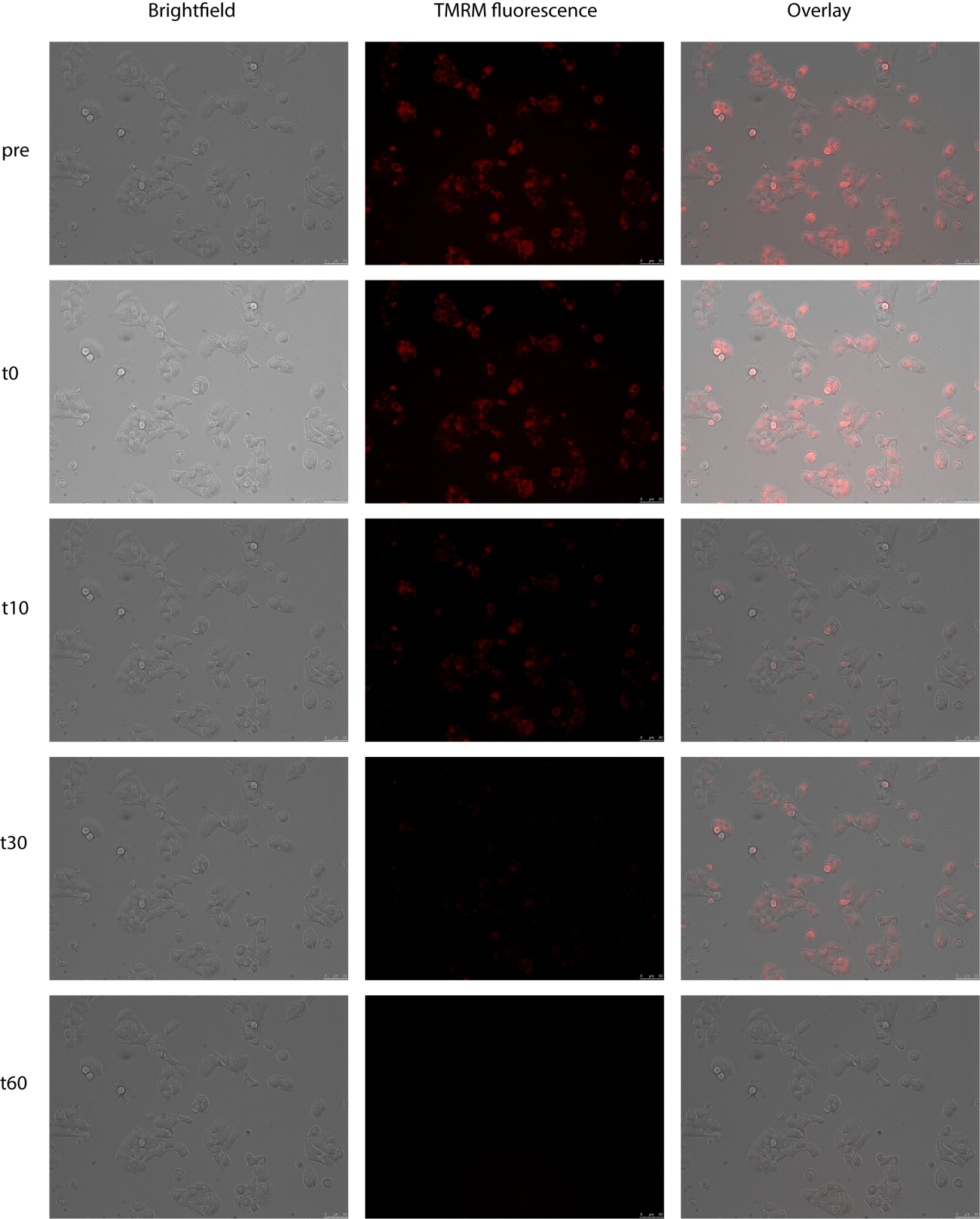


**Figure S8.** TMRM membrane potential of Colo357 cells treated with FCCP as positive control. Colo357 cells were treated with 1 µM FCCP. Pictures were taken every 10 min. Representative pictures of indicated timepoints are shown. The decreased fluorescence over time indicates a loss of mitochondrial membrane potential.


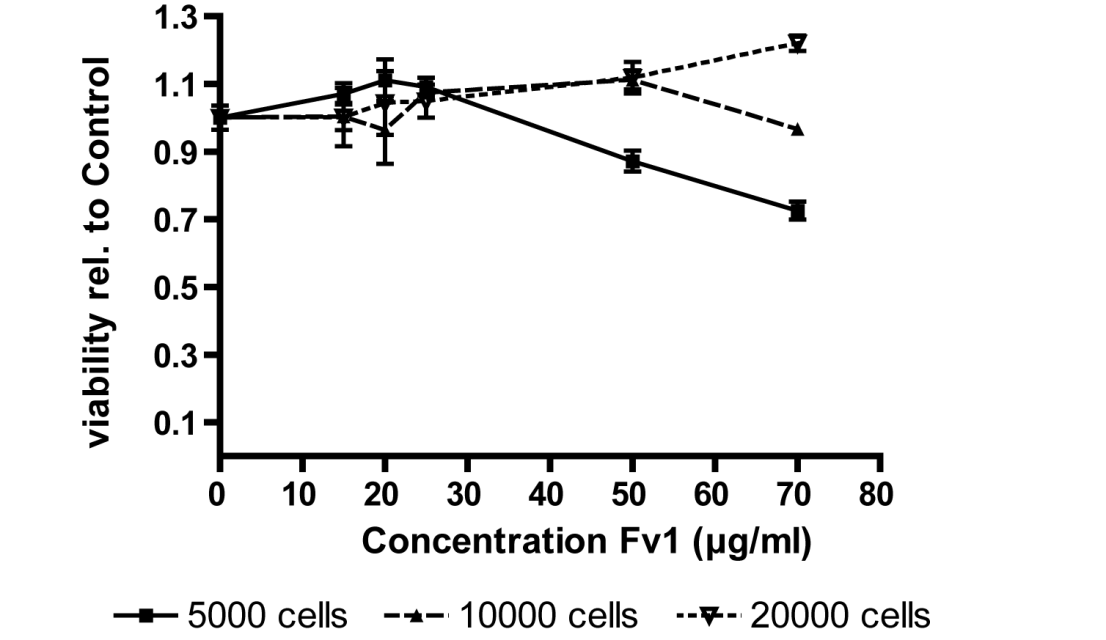


**Figure S9.** Supplement to Figure 8. Fv1 preferentially inhibits proliferating cells. A818-4 cells were seeded in different cell numbers in 96 well plates. After 24 h, they were treated with different concentrations of Fv1. 72 h after treatment, viability was measured using AlamarBlue assay.

**Table S2.** Fv1 does not induce hemolysis in fresh red blood cells. Red blood cells were isolated by careful centrifugation. 1 × 10^4^ cells were seeded in 96 well plates and treated with different concentrations of Fv1 diluted in PBS. At the indicated timepoints, the absorption was measured in the supernatant at 405/420 nm [1].

| **Concentration Fv1 (μg/mL)** | **1 h** | | **2h** | | **4 h** | |
| --- | --- | --- | --- | --- | --- | --- |
|  | **Mean** | **SD** | **Mean** | **SD** | **Mean** | **SD** |
| 0.01 | 0.0000 | 0.0026 | 0.0000 | 0.0026 | 0.0001 | 0.0023 |
| 3.13 | 0.0000 | 0.0035 | 0.0000 | 0.0023 | 0.0001 | 0.0029 |
| 6.25 | 0.0000 | 0.0026 | 0.0000 | 0.0035 | 0.0001 | 0.0026 |
| 12.5 | 0.0000 | 0.0026 | 0.0000 | 0.0023 | 0.0001 | 0.0026 |
| 25 | 0.0000 | 0.0026 | 0.0000 | 0.0029 | 0.0001 | 0.0029 |
| 50 | 0.0000 | 0.0032 | 0.0000 | 0.0040 | 0.0001 | 0.0029 |
| 100 | 0.0000 | 0.0026 | 0.0000 | 0.0021 | 0.0000 | 0.0031 |


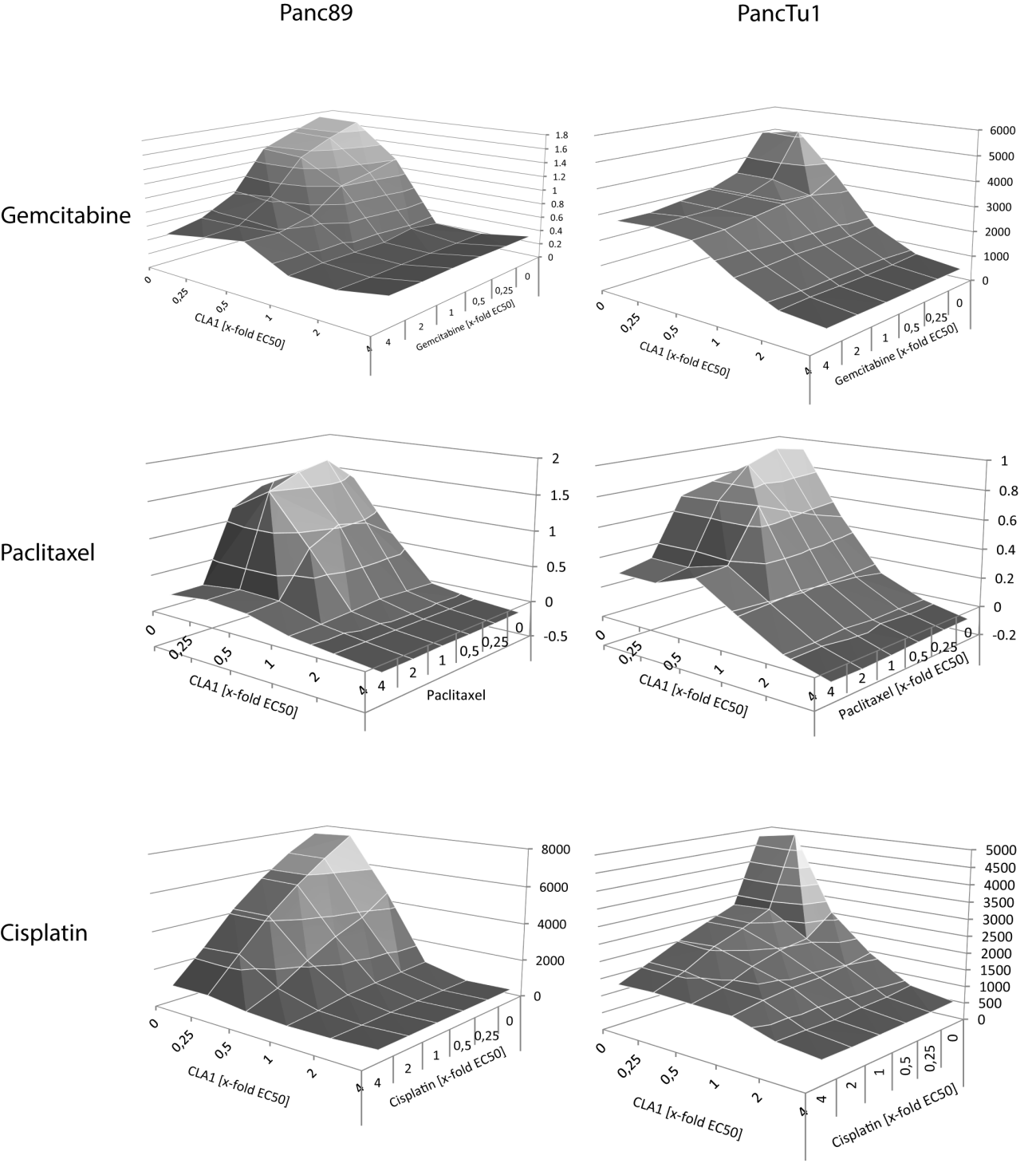


**Figure S10.** Acetonic algae extracts show additive effects in combinations with different chemotherapeutics. Panc89 and PancTu1 cells were treated with different concentrations of the algae extract CLA1 (a precursor of Fv1) and chemotherapeutics in combination. For each drug, multiple EC50 concentrations were used, which were determined by titrating single dose response curves before. Alamar Blue or XTT were used as readout and the experiment was performed in triplicates. The model of Chou [15] was used to test for synergistic or antagonistic activity of the various treatments. No synergistic or antagonistic effects were observed, which means that the substances do neither enhance nor inhibit the others effect. CLA1 was produced as follows: After the raw extract production which is described in
the methods section of the paper, the extract was diluted in 0.01% acetic acid. AMBERLITE XAD16 was added and after 2 h the extract was eluted with methanol, dried and
dissolved in DMSO.


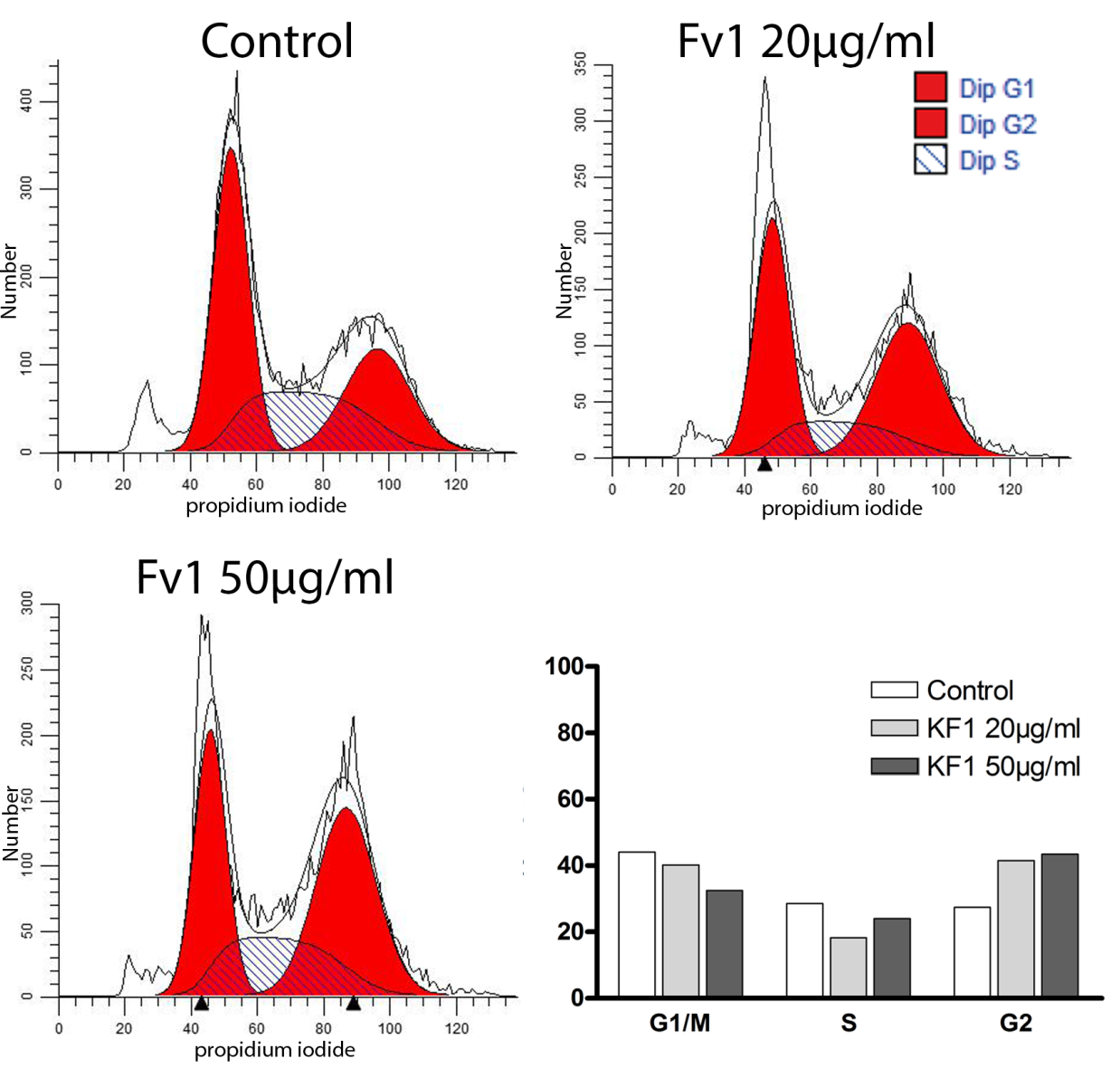


**Figure S11.** Cell cycle profile of Colo357 cells 4 h after treatment with Fv1. Experiment has been performed in parallel to Figure 4. One experiment is shown.


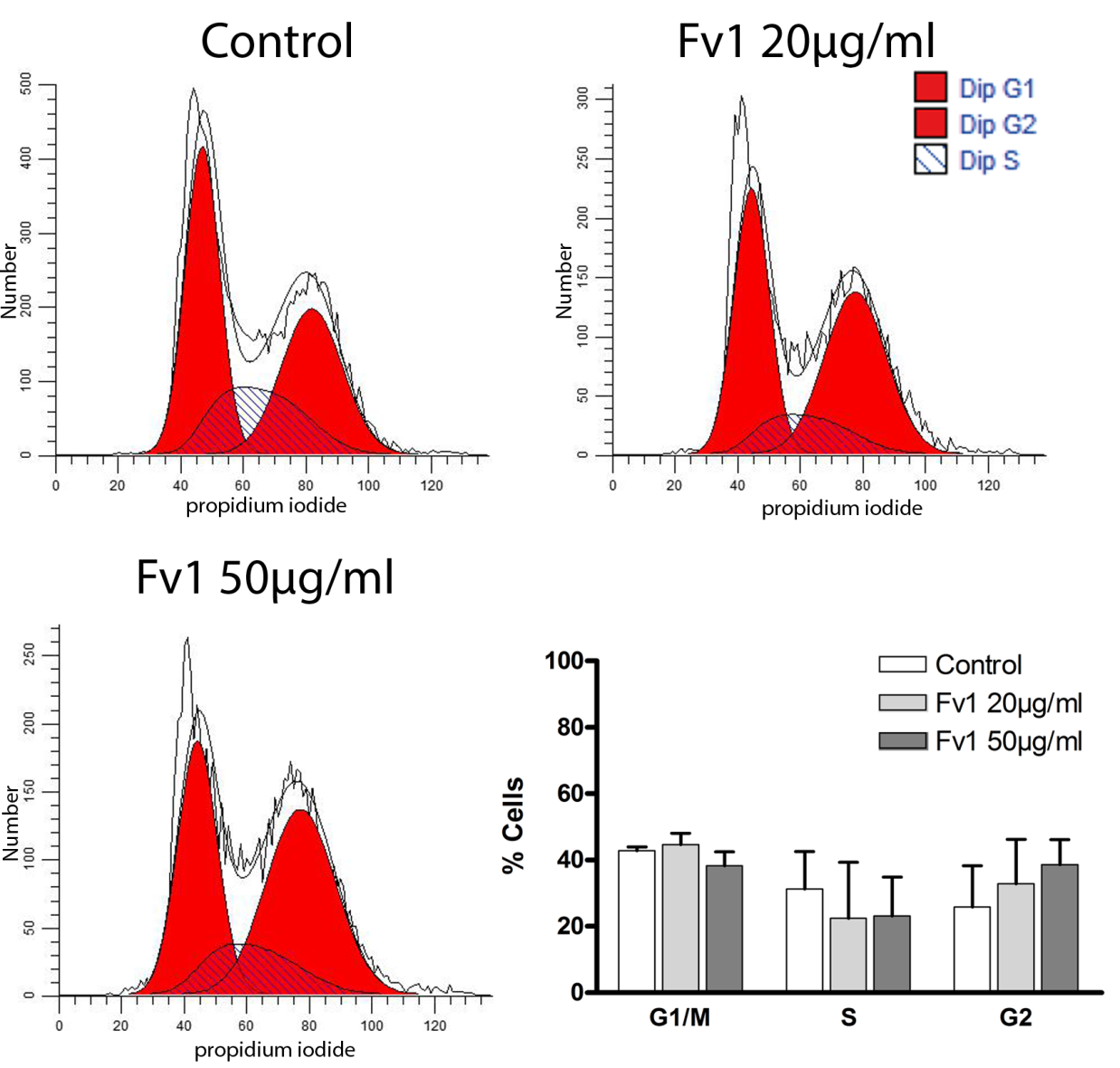


**Figure S12.** Cell cycle profile of Panc89 cells 4 h after treatment with Fv1. Experiment has been performed like described in Figure 4. Two experiments are shown in the bar chart, the histograms show one representative experiment.


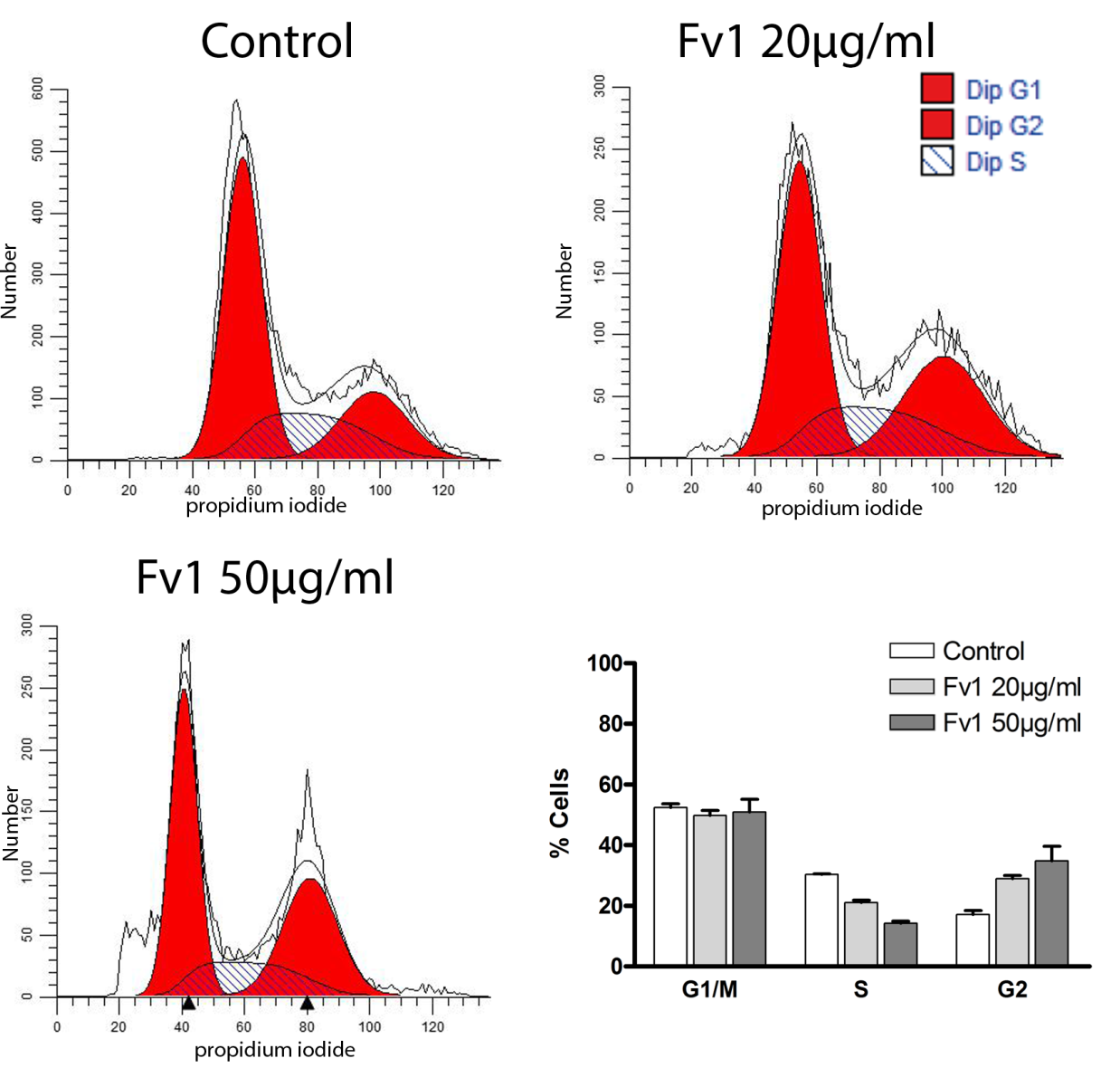


**Figure S13.** Cell cycle profile of Panc89 cells 24 h after treatment with Fv1. Experiment has been performed like described in Figure 4. Two experiments are shown in the bar chart, the histograms show one representative experiment.

**Reference**

1. Malagoli, D.A. Full-length protocol to test hemolytic activity of palytoxin on human erythrocytes. *Invertebrate Survival Journal* **2007**, *4*, 92–94.

© 2015 by the authors; licensee MDPI, Basel, Switzerland. This article is an open access article distributed under the terms and conditions of the Creative Commons Attribution license (http://creativecommons.org/licenses/by/4.0/).
